# Quantifying morphometric and adaptive characteristics of indigenous cattle genetic resources in northwest Ethiopia

**DOI:** 10.1371/journal.pone.0280640

**Published:** 2023-03-20

**Authors:** Andualem Tenagne, Mengistie Taye, Tadelle Dessie, Bekalu Muluneh, Damitie Kebede, Getinet Mekuriaw Tarekegn

**Affiliations:** 1 Department of Animal Science, College of Agriculture and Environmental Sciences, Bahir Dar University, Bahir Dar, Ethiopia; 2 Department of Animal Sciences, Assosa University, Assosa, Ethiopia; 3 Institute of Biotechnology, Bahir Dar University, Bahir Dar, Ethiopia; 4 International Livestock Research Institute (ILRI), Addis Ababa, Ethiopia; 5 Department of Animal and Range Sciences, Wolaita Sodo University, Dawuro Tarcha Campus, Tarcha, Ethiopia; 6 Animal and Veterinary Science, Scotland’s Rural College (SRUC), Roslin Institute Building, Easter Bush, Roslin, United Kingdom; Texas State University, UNITED STATES

## Abstract

Ethiopia is the gateway of livestock genetic resources to Africa and has a wide range of altitude. It is endowed with huge diverse cattle genetic resources. The aim of this research was to determine the morphometric and potentioally adaptive characteristics of cattle populations. Multi-stage purposive and random sampling methods were employed to select the study areas, households and animals. A total of 1200 adult cattle were sampled and characterized for 14 qualitative and eight morphometric variables. The comparison of marginal means, chi-square tests, canonical discriminant analysis, and clustering analysis were employed using SAS and SPSS statistical software. The sex of the animal, location and agro-ecology were fitted as fixed effects in the model and had highly significant (p<0.001) effects for most body measurements. The chi-square test values of all categorical variables were significantly different (p<0.001) and potentioally adaptive characteristics such as coat colour type, navel flap, and tail length had higher association (> 0.45) values. White with red, light red, black and dark red were the most predominant coat colour types of cattle. The maximum hit rates were recorded in Enebsie and Sinan cattle. From five extracted canonical variate, (can1 and can2) accounted 75.4% and 78.8% in the female and male cattle populations, respectively. The canonical class has separated cattle populations of Sinan from Banja at can1 and Mecha from Sinan populations at can2. The square Mahalanobis distances between sites were significant (p<0.001) and the largest distance was found between Banja and Sinan locations. Cluster analysis result classified the study populations into four major cattle groups. The cumulative analysis results showed that the cattle populations of the study area can be categorized into four breed types as Jawi Sanga, Gojjam Zenga, Banja cattle, and Sinan cattle. However, this morphology based grouping need to be confirmed by molecular data.

## Introduction

Sub-Saharan Africa region harbors about 150 native cattle breeds [1, 2]. The indigenous cattle breeds/strains are classified into nine broad groups as Humpless Longhorns, Humpless Shorthorns, Large East African Zebu, Small East African Zebu, West African Zebu, Sanga, Zenga, recently derived breeds and Commercial composites [3, 4]. Due to its geographical proximity to Near-East, Arabian, and Indian countries, Ethiopia is considered a migratory corridor of cattle into Africa [1]. Ethiopia’s agro-ecology, cultural, and ethnic diversity are believed to contribute to the maintenance of 28 recognized indigenous cattle breeds in the country [5, 6]. Cattle have significantly contributed to the livelihoods of millions of Ethiopian farmers as a source of draught power, milk, meat, manure, serve as a source of cash income and play a significant role in the socio-cultural lives of societies [7, 8]. The total number of cattle was estimated to be 70 million [9] and is the largest populous in Africa and 5^th^ in the world. About 97.4% are indigenous breeds, which are kept under extensive management, however, crossbred and exotic breeds account for only 2.3% and 0.31%, respectively [9]. This is because indigenous cattle have been naturally selected for adaptive traits such as disease tolerance and resistance, as well as adaptation to harsh environments and low-quality feeds for many years [10, 11]. Indigenous cattle breeds are more adapted to high temperatures, solar radiation, and dry conditions than exotic cattle breeds due to their short hair, thin skin, and high skin pore density, which allows them to effectively regulate their body temperature [12]. Despite the potential of diverse genetic resources, the huge loss of cattle genetic diversity in developing countries, and the contribution of cattle to food security affirmation and poverty reduction is undermined and suffer lack of due attention [13]. Even though characterization studies were conducted [14–21] in different parts of Ethiopia the characterization work remains at a rudimentary level [22]. From the identified indigenous breeds, about 62% of the status the breeds have not been not known and 34% of them was reported declining in number and at risk of extinction due to different factors [23]. Unless more reorganization and conservation work is done, half of the current cattle diversity in Africa will be lost in the next 20–50 years and the problem in Ethiopia is becoming serious [13]. In Ethiopia, only 41% of the indigenous cattle are morphologically characterized [22]. The northwestern part of Ethiopia (Gojjam area), demarcated and isolated by Abay (Blue Nile) gorge from other parts of the country, have huge cattle populations and diversified agro-ecologies. However, in the area, there is insufficient morphometric characterization study conducted. The main aim of this study was to identify the physical and adaptive characteristics of cattle populations that are found in different agro-ecological zones of northwestern Ethiopia.

## Materials and methods

### Ethics approval and consent to participants

The current study was approved by Bahir Dar University College of Agriculture and Environmental Science (BDUCAES) from ethical and technical perspectives. Consent from participant farmers in the study was obtained from each participant.

### Locations

The study was conducted in six selected sites/locations: Jawi, Enebsie Sar-Midr (Enebsie), South Achefer (Achefer), Mecha, Banja and Sinan sites of northwest Amhara, Ethiopia (Table 1). The study sites were selected in consultation with regional and zone experts of livestock development offices about the potential and distribution of the local cattle in the region.

**Table 1:** Summary of cattle population, annual temperature, rainfall, coordinate points and altitude of study sites in north-western Ethiopia.

Site	*Kebele*	Latitude	Longitude	Altitude(m.a.s.l)	Annual temp./°C	Annual	Cattle population
						RF/mm	
**Jawi**	1	11°57’18"N	36°24’48"E	995	12–40	1250	252,121
	2	11°25’38"N	36°37’06"E	1365			
	3	11°33’40"N	36°31’50"E	1171			
**Enebsie**	1	10°41’35"N	38°30’35"E	1431	10–36	900–1200	67,791
	2	10°41’41"N	38°30’40"E	1207			
	3	10°42’03"N	38°30’40"E	1271			
**Achefer**	1	11°31’17"N	36°56’19"E	2052	15–23	1450–1594	337,467
	2	11°16’36"N	36°57’52"E	2000			
**Mecha**	1	11°19’28"N	37°14’05"E	2194	23–27	1500–2200	409,502
	2	11°22’26"N	37°04’32"E	1963			
**Banja**	1	10°54’39"N	36°58’04"E	2409	7–25	2200–2560	69,156
	2	10°56’48"N	36°52’08"E	2337			
	3	10°58’36"N	37°00’55"E	3028			
**Sinan**	1	10°38’27"N	37°47’53"E	3192	0–15	900–1500	37,501
	2	10°35’03"N	37°49’43"E	3081			
	3	10°38’04"N	37°49’03"E	3214			

Source: Districts agricultural office, 2021; m a.s.l. = meter above sea level, temp = temperature in degree Celsius, and RF = annual average rainfall in millimeters.

### Sampling technique and data collection

Multi-stage sampling techniques were employed for the study. First, the study areas that are accessible for the characterization work were purposively selected and stratified into three strata based on agro-ecology as low land, midland and highland. At the next stage, two sites in each agro-ecology; Jawi and Enebsie Sar-Midr from the lowland (three *kebeles/* peasant associations from each site), South Achefer and Mecha from the midland (two *kebeles* from each site), and Banja and Sinan from highland (three *kebeles* from each site) were purposively selected based on cattle population potential. Third, farmers owning indigenous cattle were selected randomly. Finally, 1200 mature animals (800 cows and 400 bulls of age greater than 4 years), 75 animals from each *kebele* were randomly sampled for morphological characterization based on [24] guidelines. Eight linear body measurements that include mouth/muzzle circumference: the circumference (in centimeters) of the mouth immediately behind the muzzle; horn length: distance from the base of the tip of the horn to the tip of the horn; body length: the horizontal length (cm) from the point of the shoulder to the pin bone; chest girth: the distance around the animal (in centimeters) measured directly behind the front leg; height at withers: the height (in centimeters) from the bottom of the front foot to the highest point of the shoulder between the withers; pelvic width: the horizontal distance (in centimeters) between the extreme lateral points of the hook bone (tuber coxae) of the pelvis; and cannon bone circumference: the circumference (in centimeters) of the cannon bone of the foreleg of the animal were taken. Fourteen qualitative variables (body hair coat color, body hair coat colour pattern, udder size, muzzle colour, horn shape, horn orientation, hoof colour, ear orientation, hump size, navel flap (for cows), preputial, sheath (for bulls), facial profile, eyelid colour, and tail length) were recorded. Pictures of representative herds (animals) were taken using digital camera [24]. Participatory focus group discussions were used to gather further information about the origin, distribution, local name, and unique traits of indigenous cattle in each study site. The conversation of each group contained 12 members that included elders, selected cow keepers, veterinarians, and local animal production professionals.

### Data management and statistical analysis

MS Excel was used to enter, clean, and manage all of the data. Scatter plots and normality tests were used to ensure whether the quantitative variables are normal. The quantitative data were analyzed using the General Linear Model Procedures of the Statistical Analysis System (PROC GLM of SAS version 9.4) to detect phenotypic differences between sample cattle populations. The least-squares mean separation was performed using Tukey’s test of multiple comparisons [25]. The interaction of sex with agro-ecology and location/site was expressed as the least square mean (LSM). Categorical variables were subjected to the frequency procedure of Statistical Package for Social Sciences (SPSS version 21). Contingency-coefficient and phi-coefficient were the two measures of association employed to see the level of association of locations with categorical variables. The following models were used to assess the quantitative data fixing sex, location, agro-ecology, and the interactions as fixed effects in the model.


$${\text{Y}}_{\text{ijk}}=\mu +\;{\text{S}}_{\text{i}}+{\text{L}}_{\text{j}}+\;{\text{A}}_{\text{K}}+\;{\left(\text{SA}\right)}_{\text{ij}\;+}{\left(\text{SL}\right)}_{\text{ik}\;+}{\text{e}}_{\text{ijk}}$$

Where: Y_ijk_ is the observed value of the linear body measurements; μ is the overall mean;

S_i_ is the fixed effect of i^th^ sex (i = female and male), L_k_ is fixed effect location j^th^ (k = Jawi, Enebsie Sarmidr, South Achefer, Mecha, Banja Shkudad and Sinan); A_j_ is fixed effect agro-ecology j^th^ (j = low land, midland and highland); (SA)_ij_ is the interaction effect of sex with agro-ecology; (SL)_ik_ is the interaction effect of sex with location and e_ijk_ is the residual error.

Discriminant analysis was used for quantitative variables to classify the sampled populations into homogenous/distinct groups on the basis of the measured variables [15, 26]. Stepwise discriminant function analysis (STEPDISC) was used to rank the variables by their discriminating power among sample populations. Canonical discriminant function analysis (CANDISC) was performed to determine the linear combination of quantitative variables, which had maximal separations and used to check distance among populations. Non-parametric discriminant analysis was performed by merging data for both female and male samples, since there was no significant difference between sex groups for most categorical variables to check the importance of variables in classifying the sample populations. Cluster analysis was also used to classify the sampled cattle population using morphometric variables, and dendrograms were constructed for the identified groups and breed types

## Results

### Quantitative morphometric traits

The general linear model analysis demonstrated that all quantitative variables were highly (p<0.001) affected by the sex of the animal (Table 2). All quantitative variables except, cannon bone circumference showed significant difference (P<0.001) among lowland, highland and midland agro-ecologies. Similarly, there was also significant variation between locations, which are found the same agro-ecology (Table 2). For most of the measurements, the highest least squares mean were recorded for female populations in the Jawi and Mecha areas. The smallest values were recorded for cattle populations of Banja and Enebsie Sar-Midr locations. At different levels of significance, the interaction of sex and agro-ecology was significant for all quantitative measurements except mouth circumference and height. An indigenous bull from highland agro-ecology had a lower record for most of the body measurements, whereas cows found in lowland agro-ecology had higher values for most of the body measurements (Table 2). Sex and location also showed significant interactions in all body measurements at different significant levels. Bulls in Jawi and Mecha had the higher body measurements, while cows in Sinan and Enebsie had the smallest values in most measurements (Table 2).

**Table 2:** Least square means (+SE) of body measurements (cm) of cattle population in west Amhara region, Ethiopia.

Effect and levels	N	MC	HL	BL	CG	PW	HW	CBC	BW
		LSM±SE	LSM±SE	LSM±SE	LSM±SE	LSM±SE	LSM±SE	LSM±SE	LSM±SE
**Overall**	1200	36.35±0.08	20.70±0.24	111.35±0.19	138.25±0.26	32.20±0.07	111.26±0.15	19.97±0.05	213.75±1.57
**CV %**		6.69	20.85	5.7	5.74	7.54	4.19	6.92	16.52
**Sex**	**1200**	***	***	***	***	*	***	***	***
**Female**	800	35.39±0.08^b^	20. 33±0.15^b^	110.13±0.22^b^	135.86±0.28^b^	32.07±0.08^b^	110.28±0.17^b^	19.31±0.05^b^	197.31±1.31^b^
**Male**	400	38.29±0.12^a^	21.24±0.22^a^	113.84±0.31^a^	143.09±0.39^a^	32.39±0.12^a^	113.5±0.23^a^	21.29±0.07^a^	230.44±1.85^a^
**Agro-ecology**		**	***	***	***	**	***	**NS**	***
**Lowland**	450	36.40±0.12 ^b^	19.76±0.22 ^c^	113.56±0.31 ^a^	140.82±0.42 ^a^	32.44±0.12 ^a^	112.14±0.25 ^a^	20.27±0.087 ^a^	219.49±1.82^a^
**Midland**	300	36.98±0.15 ^a^	20.06±0.27 ^b^	112.44±0.38 ^a^	139.87±0.52 ^b^	32.07±0.15 ^b^	112.85±0.30 ^a^	20.22±0.09 ^a^	215.34 ±2.23^b^
**Highland**	450	37.17±0.12 ^a^	22.47±0.22 ^a^	110.54±0.31 ^b^	137.82±0.42 ^c^	32.19±0.12 ^b^	110.73±0.25 ^b^	20.39±0.07 ^a^	206.81±1.82 ^c^
**Site**		***	***	*	***	***	***	***	***
**Jawi**	225	37.04±0.16^ab^	17.51±0.29^e^	113.56±0.43^a^	145.08±0.54^a^	32.76±16^a^	114.47±0.32^a^	20.69±0.10^a^	241.16±2.26^a^
**Enebsie**	225	35.84±0.16^c^	21.56±0.29^bc^	112.46±0.43^abc^	137.01±0.524^c^	32.29±0.16^ab^	109.92±0.32^c^	19.86±0.10^b^	197.82±2.26^c^
**Achefer**	150	36.44±0.20^bc^	20.07±0.36^d^	111.32±0.52^bcd^	137.73±0.65^c^	31.71±0.20^b^	112.66±0.39^b^	19.99±0.12^b^	204.89±2.76^c^
**Mecha**	150	37.43±0.20^a^	20.50±0.36^cd^	112.97±0.52^ab^	141.48±0.65^b^	32.40±0.20^a^	112.72±0.39^b^	20.55±0.12^a^	225.79±2.76^b^
**Banja**	225	37.04±0.16^ab^	23.12±0.29^a^	110.50±0.43^d^	138.16±0.54^c^	31.67±0.16^b^	112.87±0.32^b^	19.86±0.10^b^	201.16±2.26^c^
**Sinan**	225	37.26±0.16^a^	21.97±0.29^bc^	111.09±0.43^cd^	137.39±0.54^c^	32.54±0.16^a^	108.71±0.32^c^	20.84±0.10^a^	212.45±2.26^c^
**Sex** * **agro**		**NS**	***	***	*	**	**NS**	*	*
**F, low**		35.08±0.14	18.62±0.26	111.03±0.37	137.87±0.48	32.55±0.14	110.70±0.29	19.28±0.08	205.51±2.10
**F, mid**		35.39±0.17	20.30±0.32	109.71±0.45	135.47±0.59	31.86±0.17	110.74±0.36	19.40±0.10	195.51±2.57
**F, high**		35.68±0.17	22.24±0.26	109.47±0.37	134.06±0.48	31.49±0.14	109.30±0.29	19.29±0.08	190.87±2.10
**M, low**		37.72±0.20	20.90±0.37	115.13±0.52	143.77±0.68	32.33±0.20	113.59±0.41	21.27±0.12	233.47±2.97
**M, mid**		38.58±0.25	19.82±0.45	115.13±0.63	144.27±0.84	32.29±0.24	114.97±0.50	21.04±0.15	235.17±3.64
**M, high**		38.65±0.20	22.70±0.37	111.61±0.52	141.58±0.68	32.58±0.20	112.16±0.41	21.49±0.12	222.80±2.97
**Sex** * **site**		***	*	***	***	***	***	***	***
**F, Jawi**		35.11±0.18	16.70±0.35	110.32±0.49	140.31±0.60	32.67±0.19	112.20±0.37	19.63±0.11	215.97±2.61
**F, Enebsie**		35.05±0.18	20.55±0.35	111.74±0.49	135.43±0.60	32.43±0.19	109.19±0.37	18.93±0.11	195.05±2.61
**F, Achefer**		34.94±0.22	19.66±0.43	108.72±0.61	134.36±0.74	31.71±0.23	111.27±0.45	19.27±0.14	190.89±3.19
**F, Mecha**		35.83±0.22	20.93±0.43	110.68±0.61	136.57±0.74	32.00±0.23	110.21±0.45	19.52±0.14	200.13±3.19
**F, Banja**		36.59±0.18	23.03±0.35	110.86±0.49	137.44±0.60	32.12±0.19	112.27±0.37	19.10±0.11	204.28±2.61
**F, Sinan**		34.77±0.18	21.45±0.35	108.07±0.49	130.69±0.60	31.47±0.19	106.33±37	19.47±0.11	177.46±2.61
**M, Jawi**		39.45±0.27	18.67±0.49	118.20±0.70	151.00±0.85	32.80±0.27	117.41±0.52	21.81±0.16	266.34±3.68
**M, Enebsie**		36.00±0.27	23.13±0.49	112.07±0.70	136.55±0.85	31.85±0.27	109.76±0.52	20.73±0.16	200.59±3.68
**M, Achefer**		37.98±0.31	20.44±0.61	114.66±0.86	140.86±1.04	31.54±0.33	113.82±0.64	20.44±0.20	218.90±4.51
**M, Mecha**		39.18±0.31	19.20±0.61	115.70±0.86	147.68±1.04	33.04±0.33	116.12±0.64	21.64±0.20	251.45±4.51
**M, Banja**		36.52±0.26	22.85±0.49	107.95±0.70	135.99±0.85	30.61±0.27	112.47±0.52	20.40±0.16	198.15±3.68
**M, Sinan**		40.79±0.26	22.55±0.49	115.27±0.70	147.17±0.85	34.55±0.27	111.85±0.52	22.59±0.16	247.45±3.68

Key-: CG = Chest girth; PW = pelvic width; BL = Body length; HW = Height at wither; CBC = Cannon bone circumference; MC = Mouth circumference; HL = Horn length, BW = body weight, N = number of observation, F = female, M = male, row means within cattle population, which have different superscript letter are statistically different (

* = p<0.05,

** = p<0.01,

*** = p<0.001).

### Qualitative morphometric traits

**Fig 1:**
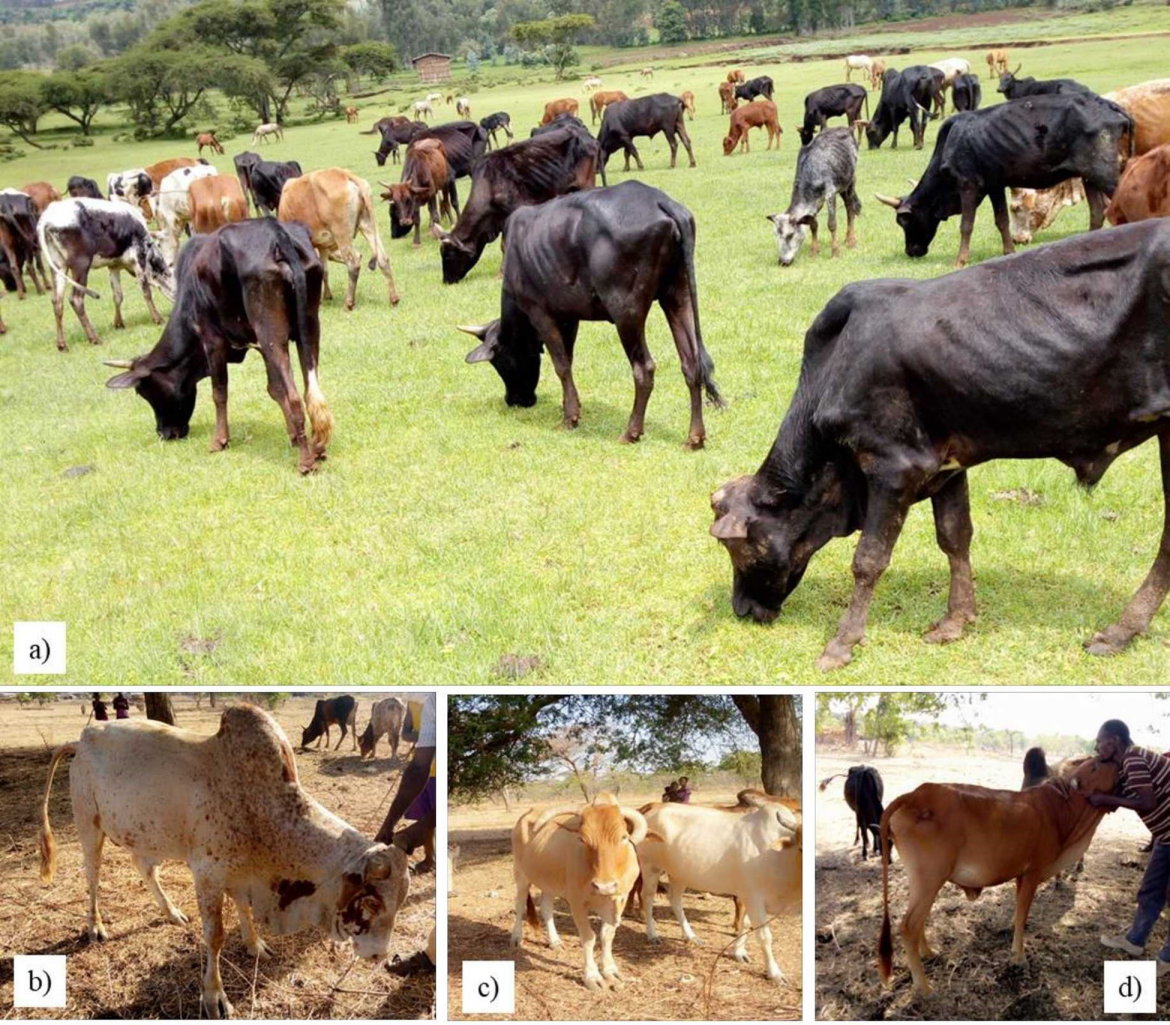
Coat colour types of Sinan cattle (a), large hump and preputial, sheath for Mecha bull (b), horn orientations and facial profile for Jawi cattle (c) and large tail length and navel flap for Jawi cow (d).

The chi-square test values of all categorical variables were presented (Table 3). The associated values ranged from 0.11 to 0.58 phi-coefficients and 0.11 to 0.51 contingency for both eyelid colour and coat colour type, respectively. Generally, coat colour type, navel flap, and tail length showed (0.58, 50, and 49) phi-coefficients and (0.51, 0.45, and 0.44) contingency coefficient values and were significantly different (p <0.001). The major qualitative results of different locations are also shown in Fig 1.

**Table 3:** Frequency percentage, chi-square test and level of association for categorical variables of cattle populations in northwest Ethiopia.

Phenotypic variables		Locations and No. of samples						Overall mean (N = 1200)	P-value	Phi coefficient	Contingency Coefficient
		Jawi (N = 225)	Enebsie (N = 225)	Achefer (N = 150)	Mecha (N = 150)	Banja (N = 225)	Sinan (N = 225)				
**Coat colour pattern**	Plain	58.7	73.3	67.3	73.3	70.7	75.6	69.8	<0.001	0.17	0.17
	Patchy	16.0	17.3	18.0	10.0	12.0	12.0	14.2			
	Spotted	25.3	9.3	14.7	16.7	17.3	12.4	16			
**Hair coat colour taype**	Black	1.3	2.7	10.0	11.3	16.4	41.2	14.3	<0.001	0.58	0.51
	Dark Red	3.6	13.3	15.3	17.3	19.6	13.3	13.4			
	Light Red	20.9	17.3	21.3	20.0	12.4	3.1	15.3			
	Fawn	9.8	18.2	6.0	6.7	12.4	2.7	9.7			
	Grey	7.6	9.8	4.7	8.0	4.4	3.6	6.3			
	White	17.8	5.3	4.0	7.3	0.4	2.2	6.3			
	White × Red	32.9	17.3	22.0	16.7	15.6	8.9	18.8			
	White × Black	5.8	9.3	10.0	9.3	11.6	15.6	10.3			
	Fawn × Cloudy	0.4	2.7	3.3	1.3	1.3	7.1	2.8			
	Brown × Black	0	4.0	3.3	2.0	5.8	1.8	2.8			
**Udder size**	Small	26.2	32.4	19.3	9.3	15.6	17.3	20.8	<0.001	0.29	0.28
	Medium	37.3	34.2	40.0	40.7	40.0	47.6	39.9			
	Large	3.1	0	7.3	16.7	11.1	1.8	6.0			
**Muzzle colour**	Pigmented	18.7	8.0	12.7	12.0	12.9	1.8	10.8	<0.001	0.18	0.18
	Non-Pigmented	81.3	92.0	87.3	88.0	87.1	98.2	89.2			
**Horn shape**	Straight	60.9	67.1	70.0	64.7	58.2	80.4	66.8	<0.001	0.33	0.31
	Curved	28.9	32.9	27.3	32.7	35.6	18.7	29.3			
	Lyre-Shape	0	0	0	0	4.4	0	0.8			
	Loose	2.7	0	0	0	0	0.9	0.7			
	Stumps	7.6	0	2.0	2.7	1.8	0	2.3			
**Horn orientation**	Lateral	53.3	44.4	53.3	49.3	32.0	51.6	46.8	<0.001	0.30	0.29
	Upward	40.0	38.2	20.7	32.0	37.3	23.1	32.6			
	Downward	2.7	0	2.7	4.0	3.1	2.2	2.3			
	Forward	3.1	13.3	19.3	14.7	25.3	23.1	16.4			
	Back Ward	0.9	4.0	3.3	0	2.2	0	1.8			
**Hoof colour**	Pigmented	12.4	21.8	11.3	14.0	10.2	10.2	13.4	<0.001	0.13	0.12
	Non Pigmented	87.6	78.2	88.7	86.0	89.8	89.8	86.			
**Ear orientation**	Erect	4.9	13.3	6.7	7.3	4.0	6.2	7.1	<0.001	0.20	0.20
	Lateral	95.1	83.6	85.3	91.3	93.3	92.9	90.5			
	Drooping	0	3.1	8.0	1.3	2.7	0.9	2.4			
**Hump size**	Small	67.6	71.1	61.3	57.3	54.7	64.9	63.2	<0.001	0.30	0.29
	Medium	11.1	27.1	22.0	28.7	42.2	26.2	26.3			
	Large	21.3	1.8	16.7	14.0	3.1	8.9	10.4			
**Preputial sheath(bull)**	Small	7.6	9.3	2.7	2.0	7.6	3.6	5.8	<0.001	0.19	0.19
	Medium	17.3	20.4	22.7	20.0	21.3	28.0	21.7			
	Large	8.4	3.6	8.0	11.3	4.4	1.8	5.8			
**Navel flap**	Small	12.4	57.3	40.7	30.7	35.6	56.6	39.2	<0.001	0.50	0.45
	Medium	41.3	5.8	20.0	29.3	27.6	2.2	20.6			
	Large	12.9	0.7	5.3	6.7	3.1	0	4.3			
**Facial (head)**	Straight	68.0	76.9	78.7	72.7	79.6	96.0	79.0	<0.001	0.28	0.27
	Concave	11.6	5.8	4.7	4.7	12.9	3.6	7.5			
	Convex	20.4	17.3	16.7	22.7	7.6	0.4	13.5			
**Eye lid colour**	Pigmented	11.6	6.7	4.0	3.3	5.3	7.1	6.7	<0.001	0.11	0.11
	Non-Pigmented	88.4	93.3	96.0	96.7	94.7	92.9	93.3			
**Tail length**	Short	0.4	0.9	0	0	0	15.1	3.1	<0.001	0.49	0.44
	Medium	20.0	40.9	28.7	20.0	22.2	58.7	32.7			
	Long	79.3	58.2	71.3	80.0	77.8	26.2	64.3			

Overall, the frequently observed color types in the study area were white with red (18.8%), light red (15.3%), black (14.3%), and dark red/brown (13.34%) from ten total recorded colour types. The dominant coat colour types for each location were white with red (32.9%), light red (20.9%) and white (17.8%) for Jawi, fawn (18.2%), light red (17.3%) and white with red (17.3%) for Enebsie Sar-midr, white with red (22%), light red (21.3%) and dark red (15.3%) for South Achefer, light red (20%), dark red (17.3%) and white with red (16.7%) for Mecha, dark red (19.6%), black (16.4%) and white with red (15.6%) for Banja, black (41.2%), white with black (15.6%) and dark red (13.3%) for Sinan locations.

### Multivariate analysis

#### Discriminant analysis

The populations of all sample locations were subjected to reclassification using discriminant analysis separately for female and male sample populations to determine the rate of correct classifications. The maximum hit rates were recorded in Enebsie (94.7%) and Sinan (60.0%) for female and, Enebsie (97.3%) and Banja (93.3%) locations for male sample populations. Whereas, minimum hit rates were recorded in sites Mecha (28%) and Achefer (50%) for the female and, Mecha (32%) and Achefer (38%) for male sample populations (Table 4). The overall classification rates (hit rate) of female and male sample populations were 59.2% and 71.8%, respectively.

**Table 4:** Number of observations and percentage classified (in bracket) in different locations for female and male sample population using discriminant analysis.

Sex	Site	Jawi	Enebsie	Achefer	Mecha	Banja	Sinan	Total
**Female**	Jawi	81(54.0)	11(7.3)	23(15.3)	17(11.3)	9(6.0)	9(6.0)	150(100)
	Enebsie	0(0.0)	142(94.7)	8(5.3)	0(0.0)	0(0.0)	0(0.0)	150(100)
	Achefer	9(9.0)	7(7.0)	50(50.0)	5(5.0)	23(23.0)	6(6.0)	100(100)
	Mecha	8(8.0)	26(26.0)	19(19.0)	28(28.0)	16(16.0)	3(3.0)	100(100)
	Banja	11(7.3)	12(8.0)	22(14.7)	10(6.7)	82(54.7)	13(8.7)	150(100)
	Sinan	20(13.3)	17(11.3)	11(7.3)	3(2.0)	8(5.3)	90(60.0)	150(100)
**Male**	Jawi	46(61.3)	3(4.0)	4(5.3)	8(10.7)	5(6.7)	9(12.0)	75 (100)
	Enebsie	1(1.3)	73(97.3)	1(1.3)	0(0.0)	0(0.0)	0(0.0)	75 (100)
	Achefer	9(18.0)	0(0.0)	19(38.0)	5(10.0)	14(28.0)	3(6.0)	50 (100)
	Mecha	13(26.0)	2(4.0)	5(10.0)	16(32.0)	7(14.0)	7(14.0)	50 (100)
	Banja	1(1.3)	0(0.0)	0(0.0)	0(0.0)	70(93.3)	4(5.3)	75 (100)
	Sinan	2(2.7)	4(5.3)	0(0.0)	0(0.0)	6(8.0)	63(84.0)	75 (100)

NB:59.2% female and 71.8% male of original grouped cases correctly classified.

#### Stepwise discriminant analysis

The stepwise discriminant analysis showed a significant difference (P<0.0001) among study locations for all quantitative variables. Based on the partial *R*^*2*^, F-static’s and Wilks’ Lambda values, the first five quantitative variables that contributed more to group discrimination for both female and male populations were chest girth, mouth circumference, body weight, canon bone circumference and height at wither. Horn length and pelvic widths showed the least power in explaining the variation between populations across locations (Table 5).

**Table 5:** Stepwise selection summary table for female and male populations.

Sex	Step	Entered	PartialR^2^	F Value	Pr > F	Wilks’ Lambda	Pr < Lambda	ASCC	Pr >ASCC
**Female**	1	CG	0.2013	40.01	<0.001	0.79873942	<0.001	0.04025212	<0.001
	2	MC	0.1428	26.43	<0.001	0.68463997	<0.001	0.06821454	<0.001
	3	BW	0.1379	25.35	<0.001	0.59020152	<0.001	0.09516398	<0.001
	4	CBC	0.1198	21.54	<0.001	0.51946971	<0.001	0.11606936	<0.001
	5	HW	0.1235	22.26	<0.001	0.45532200	<0.001	0.1379016	<0.001
	6	BL	0.0924	16.06	<0.001	0.41325710	<0.001	0.15419484	<0.001
	7	HL	0.0912	15.82	<0.001	0.37554894	<0.001	0.16893780	<0.001
	8	PW	0.0511	8.48	<0.001	0.35635309	<0.001	0.17671052	<0.001
**Male**	1	CG	0.2961	33.14	<0.001	0.70393755	<0.001	0.05921249	<0.001
	2	MC	0.2091	20.79	<0.001	0.55671466	<0.001	0.10088170	<0.001
	3	BW	0.1822	17.47	<0.001	0.45525556	<0.001	0.13433910	<0.001
	4	CBC	0.1565	14.51	<0.001	0.38398961	<0.001	0.15662397	<0.001
	5	HW	0.1562	14.44	<0.001	0.32401985	<0.001	0.18664423	<0.001
	6	BL	0.1284	11.46	<0.001	0.28241095	<0.001	0.20622413	<0.001
	7	HL	0.0707	5.91	<0.001	0.26243227	<0.001	0.21738723	<0.001
	8	PW	0.0310	2.47	0.0319	0.25430624	<0.001	0.22218911	<0.001

ASCC = Average Squared Canonical Correlation

#### Canonical discriminant analysis

**Fig 2:**
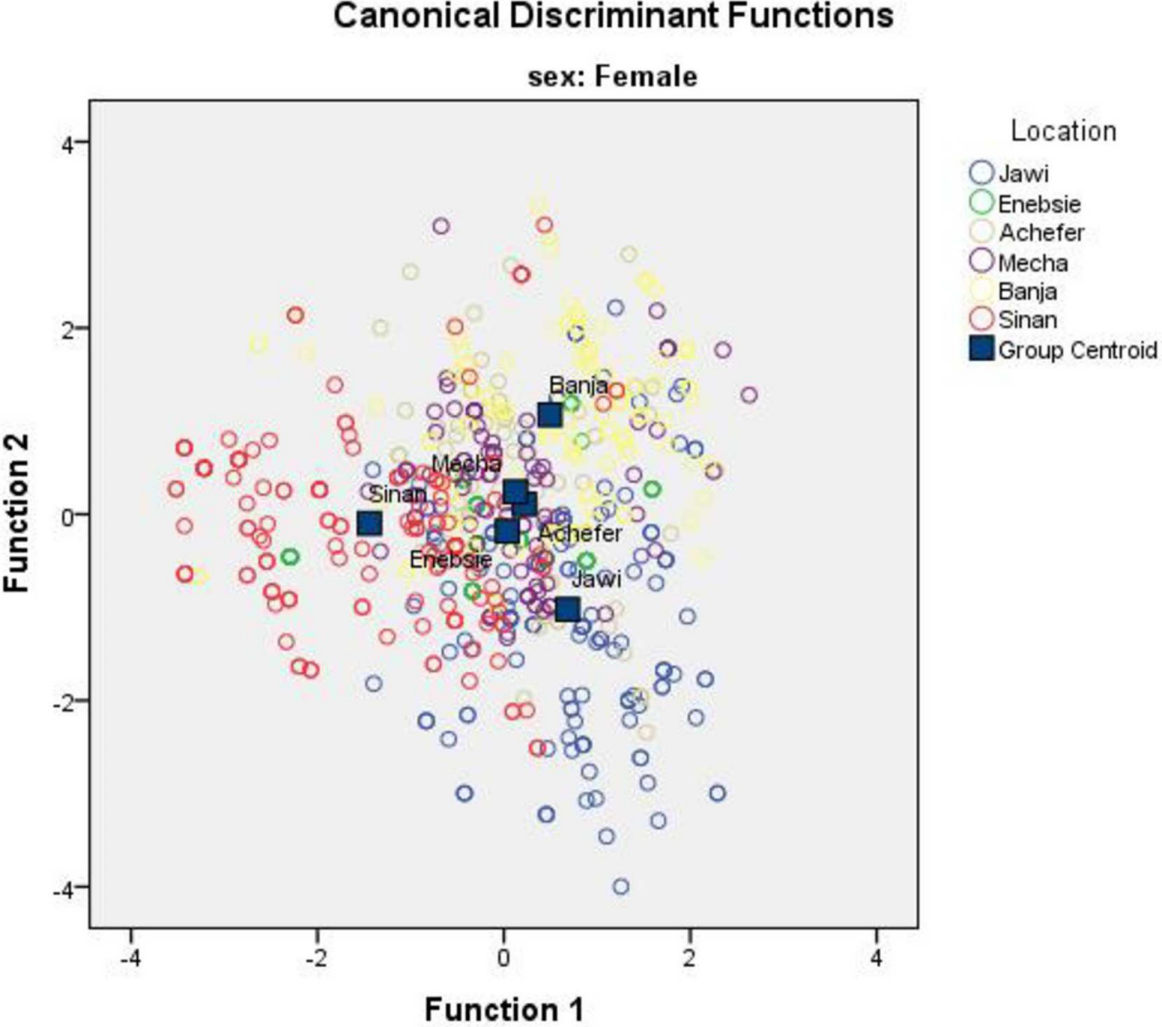
Discriminant function territorial map of morphometric variables for female sample populations.

**Fig 3:**
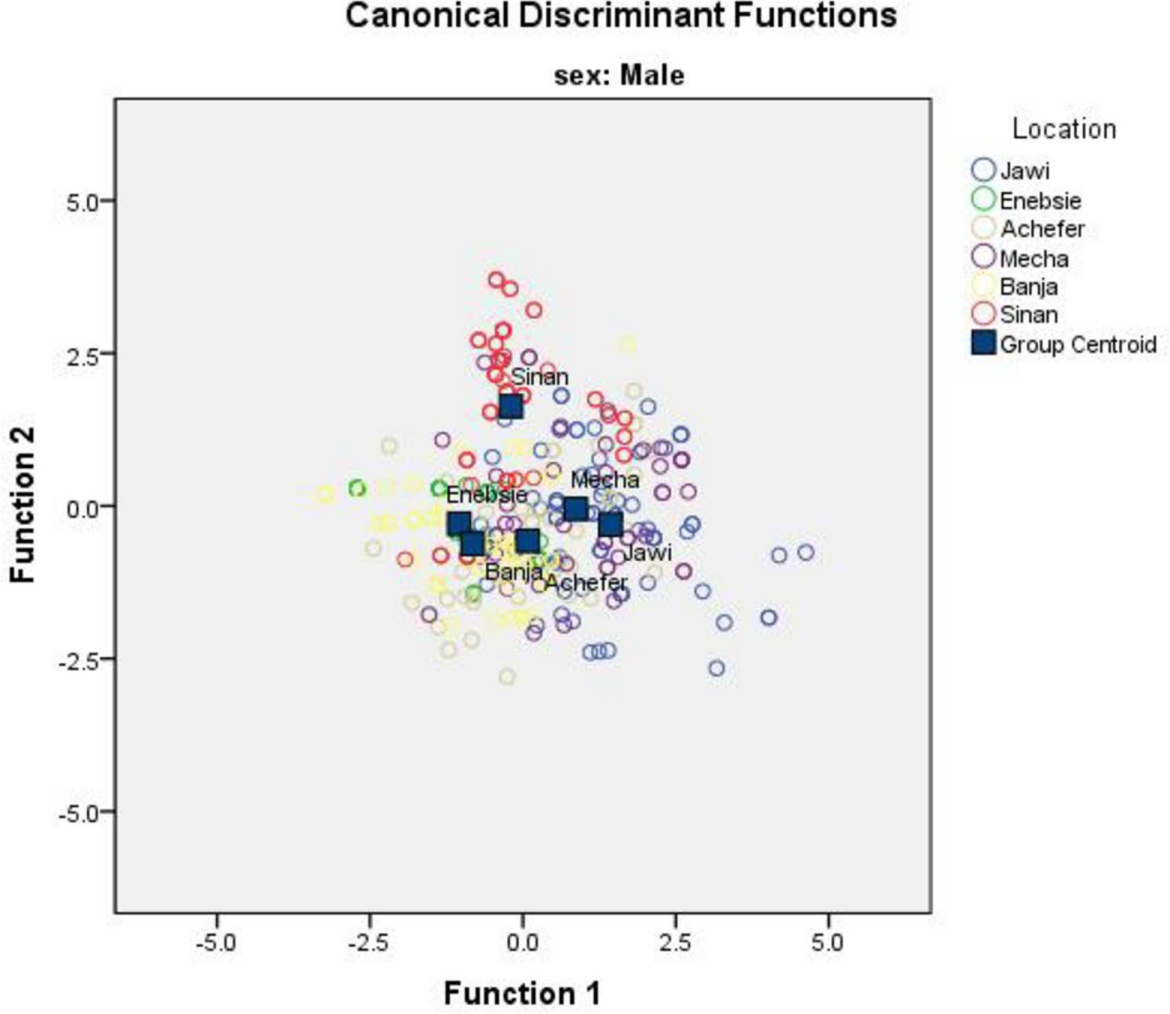
Discriminant function territorial map of morphometric variables for male sample populations.

**Fig 4:**
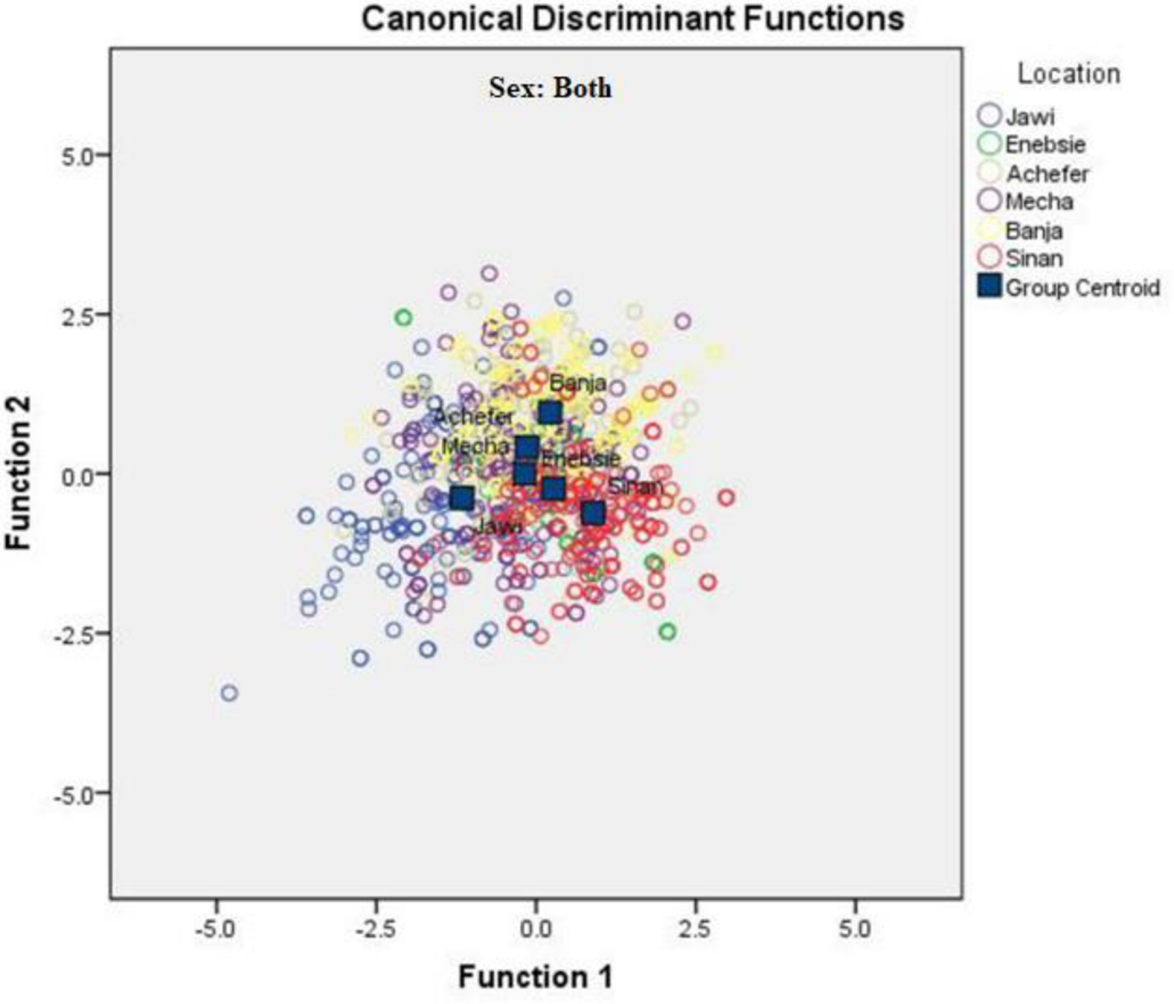
Discriminant function territorial map of morphometric variables for both sex sample populations.

Both the univariate and multivariate statistics for differences between the locations were significant (P<0.001) in the four multivariate tests (Wilks’ lambda, Pillai’s trace, Hotelling-Lawley trace, and Roy’s greatest root for female and male sample population, Table 6). Wilks’ lambda, the ratio of within-group variability to total variability on the discriminator variables, is an inverse measure of the importance of the discriminant functions. This shows that most (63.5% for female and 74.6% for male) of the variability in the discriminator variables found because of differences between populations rather than variation within populations. The highest proportion of the total cumulative variance for both female and male cattle populations were expressed by the first two canonical variates (can1 and can2) accounting for 75.4% and 78.8% in the female and male cattle populations, respectively, and the remaining three variates accounted for only 24.6% and 21.2% for female and male of the total variation (Table 6). Traits with high canonical coefficients in can1 and can2 are relatively the main contributors for characterizing the cattle populations in both sexes. Canonical discriminant function A territorial map was created by combining all morphometric measurements from both sexes and separately for each. The map result for females clearly classified Sinan, Banja, Jawi, and the remaining populations as one group (Fig 2). The map of the male population except for Sinan did not show a large difference among cattle populations (Fig 3). While the map created by combining the two sexes classified the cattle groups similarly as female, the distance was not clearly shown as female (Fig 4).

**Table 6:** Multivariate statistics and F approximations for female and male populations.

Statistic				Value	F value	Num DF	Den DF	Pr > F
**For female**								
Wilks’ Lambda				0.35635309	22.92	40	3433.2	<0.001
Pillai’s Trace				0.88355260	21.22	40	3955	<0.001
Hotelling-Lawley Trace				1.22210313	24.00	40	2347	<0.001
Roy’s Greatest Root				0.56282737	55.65	8	791	<0.001
	Eigen value	Proportion	Cum.	Ratio	F value	Num DF	Den DF	Pr > F
1	0.5628	0.4605	0.4605	0.35635309	22.92	40	3433.2	<0.001
2	0.3589	0.2937	**0.7542**	0.55691836	17.89	28	2842.6	<0.001
3	0.2076	0.1698	0.9241	0.75679858	12.84	18	2232.1	<0.001
4	0.0734	0.0601	0.9841	0.91388344	7.28	10	1580	<0.001
5	0.0194	0.0159	1.0000	0.98095962	3.84	4	791	0.0043
**For male**				**Value**	**F value**	**Num DF**	**Den DF**	**Pr > F**
Wilks’ Lambda				0.25430624	15.59	40	1689.7	<0.001
Pillai’s Trace				1.11094554	13.96	40	1955	<0.001
Hotelling-Lawley Trace				1.72832732	16.66	40	1147.1	<0.001
Roy’s Greatest Root				0.75385656	36.84	8	391	<0.001
Eigen value		Proportion	Cum.	Ratio	F value	Num DF	Den DF	Pr > F
1	0.7539	0.4362	0.4362	0.25430624	15.59	40	1689.7	<0.001
2	0.6080	0.3518	**0.7879**	0.44601666	12.55	28	1400.4	<0.001
3	0.2608	0.1553	0.9433	0.71717284	7.63	18	1100.7	<0.001
4	0.0837	0.484	0.9917	0.90971815	3.78	10	780	<0.001
5	0.0134	0.0083	1.000	0.98586894	1.40	4	391	0.2329

In spite of the variation of squared Mahalanobis distances of both sexes between each pair of the native cattle populations of study locations, the larger morphological distances were found in Banja with Sinan, Jawi with Sinan, and Jawi with Banja locations for female, respectively. In the case of male Sinan with Banja, Sinan with Jawi and Sinan with Achefer were recorded in corresponding order. The smallest distance was recorded for Mecha with Jawi in female, and Mecha with Enebsie in male cattle sample populations (Table 7).

**Table 7:** Squared Mahalanobis’ distance between locations for male (above diagonal) and female (below diagonal) sample populations.

From Site	Jawi	Enebsie	Achefer	Mecha	Banja	Sinan
**Jawi**	***	5.73327	2.38710	0.63004	5.05893	5.75819
**Enebsie**	2.26801	***	2.76978	4.38706	2.83525	6.66124
**Achefer**	1.88843	1.74935	***	1.52823	2.17718	5.63489
**Mecha**	1.64017	1.72073	0.58415	***	2.97383	3.58545
**Banja**	3.13417	4.24863	1.92470	1.20611	***	6.87150
**Sinan**	4.45108	3.74779	3.47959	2.96651	5.40164	***

According to class mean results, the first canonical variate (can1), the cattle populations of Sinan (1.31), had a higher distance from Banja (-1.08) locations in contrast to Jawi with Mecha and Enebsie with Achefer, which had relatively close distances (0.51 with 0.46) and (-0.73 with -0.46), respectively. In the second variate (can2), Mecha (1.2) showed a larger distance from the Sinan (-1.04) site, while Achefer with Mecha and Enebsie with Banja locations showed shorter distances (0.33 with 0.62) and (-0.52 with -0.28), respectively (Table 8).

**Table 8:** Class means on canonical variables of female and male population.

Sex	Site	Can1	Can2	Can3	Can4
**Female**	Jawi	0.506371781	1.200286951	0.125481179	0.172021194
	Enebsie	-0.726887891	-0.515743739	0.866794815	0.156969212
	Achefer	-0.462838863	0.328536393	0.075993463	-0.731560472
	Mecha	0.451119960	0.619231132	-0.195079763	0.075690470
	Banja	-1.083944239	-0.275401832	-0.770629249	0.151723652
	Sinan	1.312272951	-1.040986397	-0.142255877	-0.043467389
**Male**	Jawi	0.506371781	1.200286951	0.125481179	0.172021194
	Enebsie	-0.726887891	-0.515743739	0.866794815	0.156969212
	Achefer	-0.462838863	0.328536393	0.075993463	-0.731560472
	Mecha	0.451119960	0.619231132	-0.195079763	0.075690470
	Banja	-1.083944239	-0.275401832	-0.770629249	0.151723652
	Sinan	1.312272951	-1.040986397	-0.142255877	-0.043467389

#### Nonparametric discriminant analysis

The overall hit rate obtained from nonparametric discriminant classification was 54.29% (Table 9). Similar to the quantitative variable the smaller hit rates were obtained from location 3 (Achefer) and 4 (Mecha) with corresponding values (2.67 and 28.67%), in other hand larger hit rates were obtained 65.33% from 6 (Sinan) and 1 (Jawi) locations.

**Table 9:** Number of observations and percent-classified (in bracket) into site using nonparametric discriminant for both male and female sample populations.

From Site	Jawi	Enebsie	Achefer	Mecha	Banja	Sinan
**Jawi**	138(61.33)	21 (9.33)	2 (0.89)	10 (4.44)	36 (16.00)	18 (8.00)
**Enebsie**	23(10.22)	101(44.89)	2(0.89)	7(3.11)	36(16.00)	56(24.89)
**Achefer**	33(22.00	23(15.33)	4(2.67)	22(14.67)	44(29.33)	24(16.00)
**Mecha**	28(18.67)	17(11.33)	2(1.33)	43(28.67)	44(29.33)	16(10.67)
**Banja**	56(23.56)	28(12.44)	18(8.00)	44(19.56)	84(37.33)	26(11.56)
**Sinan**	3(1.33)	40(17.78)	6(2.67)	8(3.56)	23(10.22)	147(65.33)

#### Cluster analysis

**Fig 5:**
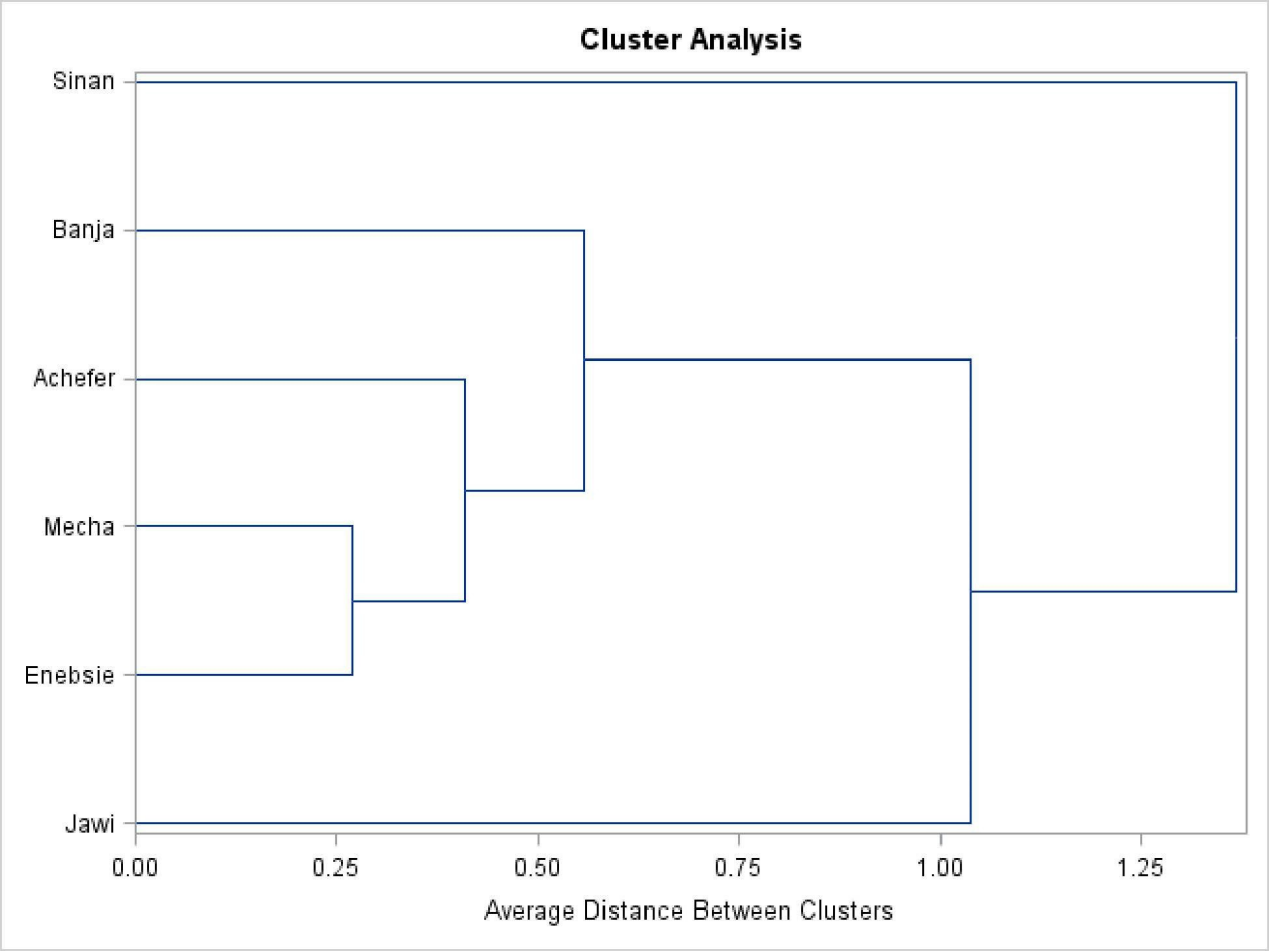
Cluster analysis result of morphometric variables for female sample populations.

**Fig 6:**
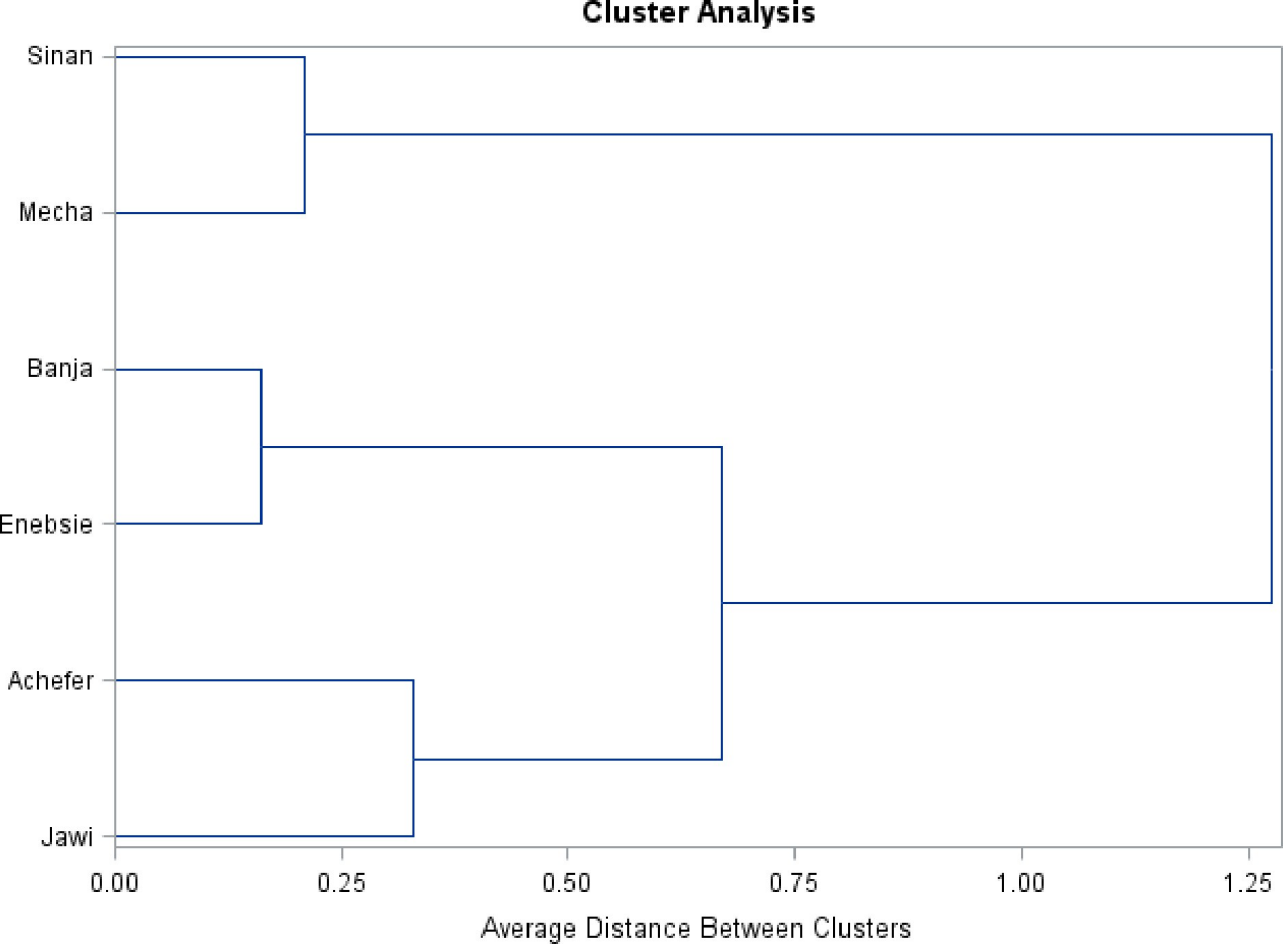
Cluster analysis result of morphometric variables for male sample populations.

Dendrograms were obtained by running cluster analysis using the Unweighted Pair-Group Method of arithmetic average distance (UPGMA) [27]. Dendrograms were constructed using all morphometric measurements for female and male sample populations, separately (Figs 5 and 6). The dendrogram result of females revealed, the existence of four different clusters of cattle populations when we cut at 0.5.

## Discussions

### Quantitative variables

Quantitative traits are more essential for characterization because of their direct association with productivity of animals and it is economically important. In this study, all the quantitative variables showed significant differences for both male and female populations among locations. Based on the result, it was reasonable to say that variations between locations were significantly attributed to these variables [24]. The linear body measurement least squares mean value difference between Jawi, Banja and Sinan cattle populations indicated their phenotypic distinctiveness than the remaining site cattle types. This significant morphological divergence between Jawi and Sinan cattle populations might be due to agro-ecological difference. whereas; the different for Banja location due geographical distance and location of Sinan is pocket that have low acess for genflow [28–30]. The height at wither, body length, and heart girth of Jawi cattle are equivalent to those of the Abigar, Raya Azebo, Afar, and Raya cattle breeds, which are all classified as African Sanga cattle [14–16]. However, the body frame traits and size of Enebsie Sarmidr, South Achefer, Mecha, Banja, and Sinan cattle populations were smaller than those of the cattle found in the Jawi location. This difference indicates that cattle populations other than Jawi might fall under same cattle sub groups. The body frame characteristics, such as body length, chest girth, pelvic width and height at wither of Sinan cattle were proximate with Goffa [21], Simada [31], Mursi [32] Gojjam [18] and Jigjga [33] cattle, which are categorized under the small east African zebu sub group [1, 34]. Whereas, the average conformation/body frame character of cattle populations in Enebsie Sarmidr, South Achefer, and Mecha locations is comparable with Ethiopian Zenga breeds such as Horro [17], Arado [20] and Fogera [18].

### Qualitative variables

Even if qualitative variables may not directly affect production traits, they contribute to adaption features and should be included in phenotypic characterisation investigations [35, 36]. From fourteen qualitative variables, coat colour type, navel flap and tail length had larger association values in respective order. These variables with larger associations played an important role in distinguishing cattle populations [37]. The coat colour is an important morphological trait that imparts adaptive ability of heat-stressed livestock [12]. White with red, light red, white, fawn and grey dominant coat colour of Jawi cattle were lining to the report of [21] for Goffa cattle. Jawi cattle have 89% shine/bright color type, which is similar to Boran and Ogaden cattle breeds that have successfully adapted to hot and arid settings [33, 38]. That coat color type may obtain continuous natural and human selection enabling to adapt to the hot and dry existing environment. For instance, white/light colour animals reflect 50–60% of direct solar radiation compared with the dark colour one that are advantageous in hot tropical regions [12, 39]. Sinan cattle have a black dominant coat color type, similar to Arsi and Simada cattle breeds [34], which are well-adapted to Ethiopia’s highlands [31]. The result of Sinan cattle coat colour can be of genetic origin and results from the adaptation of cattle population to adapt to cold temperatures of highland areas by absorbing the direct solar radiation [4]. The naval flap size of Jawi was mainly medium to large exceptionally from five populations included in this study and other Ethiopian cattle reported for Ogaden cattle [33], Arado, Abergelle, and Irob cattle [20] and Mursi cattle [32]. This unique naval flap size of Jawi may develop through continuous selection, hence farmers in the study location perceive animals, which have large navel flaps as better breeding animal. Tail length of Jawi cattle was mainly longer than other sites included in this study and in line with the report of Horro, Ogaden, Mursi and Boran cattle [12, 17, 19, 32, 33]. This Long tail for Jawi cattle is used to protect from biting of flies and other external parasites [12]. The overall study result of small and medium hump sizes was similar to the report of other indigenous cattle populations [19] for cattle population in North-Central Ethiopia and [20] for northern Ethiopia cattle breeds. Laterally oriented ears and straight faces for all six locations cattle populations are also the characteristics of indigenous cattle populations in North Central and South Western Ethiopia [21, 32]. Those variations in both continuous and qualitative variables provide facts about the bred differences among locations before multivariate analyses were performed [40, 41].

### Multivariate analysis

Multivariate analysis was used to quantify the level of uniqueness for each population because multivariate analyses of variance were used for assessing the aggregate morphological characteristics needed for grouping [24]. The higher classification percentages on discriminant analysis were recorded on Enebsie and Sinan cattle for female and Banja and Sinan locations in case of male sample. The high classification rate was an indicator of that homogeneous and distinctiveness of cattle populations [42]. While Mecha sites had the lowest classification percentages, this also indicated that the cattle populations in these samples were less homogeneous and shared a high number of cross-classifications with the south Achefer population. This also confirms that populations from these locations were phenotypically closely related to each other location [20, 37, 43]. The higher morphological distances were observed between Banja and Sinan cattle populations based on class means and squared Mahalanobis distance results of multivariate analysis. This result confirms the existence of the two cattle populations in different breed groups [24].

Cluster analysis is most useful for grouping sample populations into clusters that have similar features to those of other clusters [15]. The Dendrogram results of morphometric measurements for female cattle revealed the existence of four different clusters and supported with participatory focus group discussions, other multivariate and univariate analysis results. Both cluster and discriminant are the aggregate morphological variation is a linear combination of the individual variables recorded from operational taxonomic units (OTUs) [44]. However, Dendrogram result of the male sample was different and unsupported with other results. Accordingly, the final classification was determined based on the cumulative result. The first cluster consists of Jawi location (Jawi Sanga), the second cluster consists of three locations, Enebsie, Achefer, and Mecha (Gojjam zebu/zenga, which shows Zenga subclass characters), the third cluster consists of Banja location (Banja cattle that show somewhat of the Zenga and High Land Zebu subclass characters), and the fourth cluster consists of Sinan location (Sinan cattle that have the character of the Small East African Zebu subclass).

## Conclusions

All morphometric characteristics showed a significant difference among locations. Even if the variation in some morphometric measurements, such as cannon bone circumference and horn length, was not large enough to indicate the existence of distinct cattle types or breeds, the morphological divergences of most measurements indicated the existence of distinct cattle types or breeds. Qualitative traits are as important as quantitative traits for animal identification. Coat colour type, navel flap, and tail length were important categorical characters that had larger association values used for breed identification. Based on the discriminant classification, canonical correlations, and cluster analysis results, the level of uniqueness was quantified into four cattle groups. Accordingly, the cumulative analysis results showed the existence of four cattle groups. The cattle populations of the study area can be categorized into four population types as Jawi Sanga, Gojjam Zenga, Banja cattle, and Sinan cattle. However, the results of morphological classifications in this study need to be confirmed with molecular characterization to validate genetic breed differences.

## Supporting information

S1 File Raw data for qualitative morphometric used in indeginous cattle characterization. (XLSX)

S2 File The GLM SAS resuts of morphometric body measurements for sex vs location. (MHT)

S3 File The GLM SAS resuts of morphometric body measurements for sex vs location. (MHT)

S4 File Discriminant function territorial map of morphometric variables spss result for female and male sample populations. (DOC)

S5 File Discriminant function territorial map of morphometric variables spss result for two sexs by merging together sample populations. (DOC)
