# Supplementary material for: Quantifying morphometric and adaptive characteristics of indigenous cattle genetic resources in northwest Ethiopia
Source: PLoS One. 2023 Mar 20;18(3):e0280640. doi: 10.1371/journal.pone.0280640 (PMC10027228; doi:10.1371/journal.pone.0280640)
Supplement: S4 File — (DOC) [file pone.0280640.s004.doc]

SORT CASES  BY sex.
SPLIT FILE LAYERED BY sex.
DISCRIMINANT
  /GROUPS=Location(1 6)
  /VARIABLES=MC HL BL HG PW HW CBC BW
  /ANALYSIS ALL
  /PRIORS EQUAL
  /STATISTICS=UNIVF GCOV TCOV
  /PLOT=COMBINED SEPARATE  MAP
  /CLASSIFY=NONMISSING POOLED.


Discriminant


Notes	
Output Created	09-DEC-2022 17:23:22	
Comments		
Input	Data	C:\Users\DEMSSIE\Desktop\data final\Untitled3.sav	
	Active Dataset	DataSet1	
	Filter	<none>	
	Weight	<none>	
	Split File	sex	
	N of Rows in Working Data File	1200	
Missing Value Handling	Definition of Missing	User-defined missing values are treated as missing in the analysis phase.	
	Cases Used	In the analysis phase, cases with no user- or system-missing values for any predictor variable are used. Cases with user-, system-missing, or out-of-range values for the grouping variable are always excluded.	
Syntax	DISCRIMINANT
  /GROUPS=Location(1 6)
  /VARIABLES=MC HL BL HG PW HW CBC BW
  /ANALYSIS ALL
  /PRIORS EQUAL
  /STATISTICS=UNIVF GCOV TCOV
  /PLOT=COMBINED SEPARATE  MAP
  /CLASSIFY=NONMISSING POOLED.	
Resources	Processor Time	00:00:02.58	
	Elapsed Time	00:00:02.67	


[DataSet1] C:\Users\DEMSSIE\Desktop\data final\Untitled3.sav


Analysis Case Processing Summary	
sex	Unweighted Cases	N	Percent	
Male	Valid	400	100.0	
	Excluded	Missing or out-of-range group codes	0	.0	
		At least one missing discriminating variable	0	.0	
		Both missing or out-of-range group codes and at least one missing discriminating variable	0	.0	
		Total	0	.0	
	Total	400	100.0	
Femal	Valid	800	100.0	
	Excluded	Missing or out-of-range group codes	0	.0	
		At least one missing discriminating variable	0	.0	
		Both missing or out-of-range group codes and at least one missing discriminating variable	0	.0	
		Total	0	.0	
	Total	800	100.0	


Group Statistics	
sex	Location	Valid N (listwise)	
		Unweighted	Weighted	
Male	Jawi	MC	75	75.000	
		HL	75	75.000	
		BL	75	75.000	
		HG	75	75.000	
		PW	75	75.000	
		HW	75	75.000	
		CBC	75	75.000	
		 BW	75	75.000	
	Enebsie	MC	75	75.000	
		HL	75	75.000	
		BL	75	75.000	
		HG	75	75.000	
		PW	75	75.000	
		HW	75	75.000	
		CBC	75	75.000	
		 BW	75	75.000	
	Achefer	MC	50	50.000	
		HL	50	50.000	
		BL	50	50.000	
		HG	50	50.000	
		PW	50	50.000	
		HW	50	50.000	
		CBC	50	50.000	
		 BW	50	50.000	
	Mecha	MC	50	50.000	
		HL	50	50.000	
		BL	50	50.000	
		HG	50	50.000	
		PW	50	50.000	
		HW	50	50.000	
		CBC	50	50.000	
		 BW	50	50.000	

Group Statistics	
sex	Location	Valid N (listwise)	
		Unweighted	Weighted	
Male	Banja	MC	75	75.000	
		HL	75	75.000	
		BL	75	75.000	
		HG	75	75.000	
		PW	75	75.000	
		HW	75	75.000	
		CBC	75	75.000	
		 BW	75	75.000	
	Sinan	MC	75	75.000	
		HL	75	75.000	
		BL	75	75.000	
		HG	75	75.000	
		PW	75	75.000	
		HW	75	75.000	
		CBC	75	75.000	
		 BW	75	75.000	
	Total	MC	400	400.000	
		HL	400	400.000	
		BL	400	400.000	
		HG	400	400.000	
		PW	400	400.000	
		HW	400	400.000	
		CBC	400	400.000	
		 BW	400	400.000	
Femal	Jawi	MC	150	150.000	
		HL	150	150.000	
		BL	150	150.000	
		HG	150	150.000	
		PW	150	150.000	
		HW	150	150.000	
		CBC	150	150.000	
		 BW	150	150.000	

Group Statistics	
sex	Location	Valid N (listwise)	
		Unweighted	Weighted	
Femal	Enebsie	MC	150	150.000	
		HL	150	150.000	
		BL	150	150.000	
		HG	150	150.000	
		PW	150	150.000	
		HW	150	150.000	
		CBC	150	150.000	
		 BW	150	150.000	
	Achefer	MC	100	100.000	
		HL	100	100.000	
		BL	100	100.000	
		HG	100	100.000	
		PW	100	100.000	
		HW	100	100.000	
		CBC	100	100.000	
		 BW	100	100.000	
	Mecha	MC	100	100.000	
		HL	100	100.000	
		BL	100	100.000	
		HG	100	100.000	
		PW	100	100.000	
		HW	100	100.000	
		CBC	100	100.000	
		 BW	100	100.000	
	Banja	MC	150	150.000	
		HL	150	150.000	
		BL	150	150.000	
		HG	150	150.000	
		PW	150	150.000	
		HW	150	150.000	
		CBC	150	150.000	
		 BW	150	150.000	

Group Statistics	
sex	Location	Valid N (listwise)	
		Unweighted	Weighted	
Femal	Sinan	MC	150	150.000	
		HL	150	150.000	
		BL	150	150.000	
		HG	150	150.000	
		PW	150	150.000	
		HW	150	150.000	
		CBC	150	150.000	
		 BW	150	150.000	
	Total	MC	800	800.000	
		HL	800	800.000	
		BL	800	800.000	
		HG	800	800.000	
		PW	800	800.000	
		HW	800	800.000	
		CBC	800	800.000	
		 BW	800	800.000	


Tests of Equality of Group Means	
sex	Wilks' Lambda	F	df1	df2	Sig.	
Male	MC	.718	30.941	5	394	.000	
	HL	.860	12.798	5	394	.000	
	BL	.787	21.294	5	394	.000	
	HG	.704	33.142	5	394	.000	
	PW	.798	19.960	5	394	.000	
	HW	.770	23.527	5	394	.000	
	CBC	.763	24.534	5	394	.000	
	 BW	.707	32.597	5	394	.000	
Femal	MC	.887	20.193	5	794	.000	
	HL	.802	39.100	5	794	.000	
	BL	.952	8.034	5	794	.000	
	HG	.799	40.013	5	794	.000	
	PW	.964	5.977	5	794	.000	
	HW	.799	39.897	5	794	.000	
	CBC	.964	5.942	5	794	.000	
	 BW	.802	39.156	5	794	.000	


Covariance Matricesa,b	
sex	Location	MC	HL	BL	HG	PW	HW	
Male	Jawi	MC	11.819	6.761	19.476	28.811	6.659	11.121	
		HL	6.761	26.550	13.473	27.162	3.162	8.842	
		BL	19.476	13.473	60.459	66.986	14.554	28.470	
		HG	28.811	27.162	66.986	124.568	22.027	48.851	
		PW	6.659	3.162	14.554	22.027	7.378	8.259	
		HW	11.121	8.842	28.470	48.851	8.259	26.165	
		CBC	3.180	2.234	7.268	11.392	2.719	4.321	
		 BW	142.138	127.592	321.896	621.318	105.684	244.884	
	Enebsie	MC	4.027	5.324	5.892	12.581	.824	5.973	
		HL	5.324	21.306	13.923	24.305	2.006	3.951	
		BL	5.892	13.923	16.279	24.206	2.456	5.111	
		HG	12.581	24.305	24.206	56.900	1.527	20.498	
		PW	.824	2.006	2.456	1.527	1.505	-1.009	
		HW	5.973	3.951	5.111	20.498	-1.009	13.752	
		CBC	.257	1.320	1.464	.418	.352	-.605	
		 BW	49.618	94.618	95.518	226.181	5.914	81.769	
	Achefer	MC	6.673	1.070	15.116	19.120	5.297	6.200	
		HL	1.070	36.986	7.010	3.512	1.533	8.448	
		BL	15.116	7.010	52.923	46.543	12.534	16.693	
		HG	19.120	3.512	46.543	78.000	18.934	24.546	
		PW	5.297	1.533	12.534	18.934	6.376	7.181	
		HW	6.200	8.448	16.693	24.546	7.181	20.396	
		CBC	2.682	.129	6.887	10.410	3.125	3.020	
		 BW	81.239	11.681	196.684	331.134	80.682	102.095	
	Mecha	MC	9.293	3.229	18.116	32.181	8.033	13.366	
		HL	3.229	20.000	16.000	12.433	3.890	6.894	
		BL	18.116	16.000	62.949	75.371	18.053	42.220	
		HG	32.181	12.433	75.371	146.263	32.911	63.121	
		PW	8.033	3.890	18.053	32.911	9.631	12.791	
		HW	13.366	6.894	42.220	63.121	12.791	47.496	
		CBC	4.005	1.727	8.910	16.433	3.851	7.309	
		 BW	151.350	55.000	350.761	682.278	154.361	295.698	
	Banja	MC	3.956	1.929	9.406	8.021	2.650	4.295	

Covariance Matricesa,b	
sex	Location	CBC	 BW	
Male	Jawi	MC	3.180	142.138	
		HL	2.234	127.592	
		BL	7.268	321.896	
		HG	11.392	621.318	
		PW	2.719	105.684	
		HW	4.321	244.884	
		CBC	2.884	54.659	
		 BW	54.659	3131.612	
	Enebsie	MC	.257	49.618	
		HL	1.320	94.618	
		BL	1.464	95.518	
		HG	.418	226.181	
		PW	.352	5.914	
		HW	-.605	81.769	
		CBC	.333	1.363	
		 BW	1.363	899.815	
	Achefer	MC	2.682	81.239	
		HL	.129	11.681	
		BL	6.887	196.684	
		HG	10.410	331.134	
		PW	3.125	80.682	
		HW	3.020	102.095	
		CBC	2.170	45.014	
		 BW	45.014	1410.435	
	Mecha	MC	4.005	151.350	
		HL	1.727	55.000	
		BL	8.910	350.761	
		HG	16.433	682.278	
		PW	3.851	154.361	
		HW	7.309	295.698	
		CBC	2.235	76.919	
		 BW	76.919	3202.185	
	Banja	MC	2.438	34.677	

Covariance Matricesa,b	
sex	Location	MC	HL	BL	HG	PW	HW	
Male	Banja	HL	1.929	18.911	-5.805	-7.745	-2.436	-8.241	
		BL	9.406	-5.805	56.024	38.432	15.857	26.404	
		HG	8.021	-7.745	38.432	41.581	13.062	17.601	
		PW	2.650	-2.436	15.857	13.062	6.240	8.575	
		HW	4.295	-8.241	26.404	17.601	8.575	21.036	
		CBC	2.438	.357	5.049	4.789	1.711	3.703	
		 BW	34.677	-30.191	155.499	171.920	53.166	72.679	
	Sinan	MC	12.359	6.348	13.801	28.483	4.780	9.752	
		HL	6.348	5.927	7.163	15.363	1.197	6.365	
		BL	13.801	7.163	21.820	37.818	8.623	16.567	
		HG	28.483	15.363	37.818	86.010	19.350	26.485	
		PW	4.780	1.197	8.623	19.350	10.386	3.608	
		HW	9.752	6.365	16.567	26.485	3.608	21.965	
		CBC	3.586	1.905	5.558	10.302	3.175	5.155	
		 BW	126.892	69.314	170.547	385.758	85.991	121.568	
	Total	MC	11.037	2.807	17.613	30.171	6.432	10.494	
		HL	2.807	23.806	3.425	4.661	.914	-.728	
		BL	17.613	3.425	54.492	64.495	14.577	27.231	
		HG	30.171	4.661	64.495	120.551	22.656	43.755	
		PW	6.432	.914	14.577	22.656	8.393	6.595	
		HW	10.494	-.728	27.231	43.755	6.595	30.796	
		CBC	3.907	.899	7.469	12.371	3.371	4.230	
		 BW	136.303	18.876	288.516	546.619	101.779	200.237	
Femal	Jawi	MC	3.591	3.410	8.910	7.502	1.286	2.554	
		HL	3.410	22.762	7.553	8.066	2.201	.960	
		BL	8.910	7.553	61.736	35.337	8.591	13.452	
		HG	7.502	8.066	35.337	48.040	8.902	18.918	
		PW	1.286	2.201	8.591	8.902	3.633	3.477	
		HW	2.554	.960	13.452	18.918	3.477	16.295	
		CBC	.707	1.632	4.201	3.874	1.076	1.874	
		 BW	32.472	34.018	151.405	206.248	37.466	81.358	
	Enebsie	MC	2.783	-.844	2.797	4.584	1.309	-3.593	
		HL	-.844	10.236	-2.528	-6.037	.956	-4.979	

Covariance Matricesa,b	
sex	Location	CBC	 BW	
Male	Banja	HL	.357	-30.191	
		BL	5.049	155.499	
		HG	4.789	171.920	
		PW	1.711	53.166	
		HW	3.703	72.679	
		CBC	2.054	21.182	
		 BW	21.182	716.601	
	Sinan	MC	3.586	126.892	
		HL	1.905	69.314	
		BL	5.558	170.547	
		HG	10.302	385.758	
		PW	3.175	85.991	
		HW	5.155	121.568	
		CBC	3.597	46.555	
		 BW	46.555	1735.673	
	Total	MC	3.907	136.303	
		HL	.899	18.876	
		BL	7.469	288.516	
		HG	12.371	546.619	
		PW	3.371	101.779	
		HW	4.230	200.237	
		CBC	2.866	56.283	
		 BW	56.283	2500.707	
Femal	Jawi	MC	.707	32.472	
		HL	1.632	34.018	
		BL	4.201	151.405	
		HG	3.874	206.248	
		PW	1.076	37.466	
		HW	1.874	81.358	
		CBC	1.618	15.923	
		 BW	15.923	889.161	
	Enebsie	MC	.540	18.049	
		HL	-.738	-23.887	

Covariance Matricesa,b	
sex	Location	MC	HL	BL	HG	PW	HW	
Femal	Enebsie	BL	2.797	-2.528	8.153	4.550	3.415	1.467	
		HG	4.584	-6.037	4.550	15.120	1.147	.211	
		PW	1.309	.956	3.415	1.147	2.153	.151	
		HW	-3.593	-4.979	1.467	.211	.151	25.177	
		CBC	.540	-.738	1.430	1.468	.509	-.294	
		 BW	18.049	-23.887	18.058	59.171	4.419	.497	
	Achefer	MC	1.794	1.787	1.115	.284	.164	.158	
		HL	1.787	24.873	2.443	-2.018	-.534	-5.988	
		BL	1.115	2.443	13.654	3.846	.729	.498	
		HG	.284	-2.018	3.846	18.637	1.570	6.033	
		PW	.164	-.534	.729	1.570	2.693	2.534	
		HW	.158	-5.988	.498	6.033	2.534	10.401	
		CBC	.542	.295	.963	1.669	.857	1.371	
		 BW	1.157	-8.126	15.502	74.150	6.031	23.804	
	Mecha	MC	1.314	1.069	2.884	4.542	.737	.006	
		HL	1.069	20.591	10.644	7.323	2.586	-2.187	
		BL	2.884	10.644	32.886	22.841	6.545	1.482	
		HG	4.542	7.323	22.841	34.753	6.303	6.798	
		PW	.737	2.586	6.545	6.303	3.212	1.808	
		HW	.006	-2.187	1.482	6.798	1.808	14.834	
		CBC	1.069	2.229	5.602	6.731	1.485	1.092	
		 BW	18.371	28.682	92.232	140.256	25.158	27.135	
	Banja	MC	3.076	.837	8.808	10.103	2.090	4.769	
		HL	.837	16.617	3.896	1.364	.158	.476	
		BL	8.808	3.896	44.376	35.190	7.540	20.293	
		HG	10.103	1.364	35.190	61.604	10.866	32.969	
		PW	2.090	.158	7.540	10.866	3.945	6.216	
		HW	4.769	.476	20.293	32.969	6.216	26.318	
		CBC	1.216	-.607	4.625	7.701	1.807	4.477	
		 BW	41.491	6.220	145.985	253.583	44.748	135.621	
	Sinan	MC	6.458	3.533	10.641	8.116	5.214	5.982	
		HL	3.533	11.243	6.503	4.573	5.163	5.284	
		BL	10.641	6.503	31.867	20.399	10.999	15.754	

Covariance Matricesa,b	
sex	Location	CBC	 BW	
Femal	Enebsie	BL	1.430	18.058	
		HG	1.468	59.171	
		PW	.509	4.419	
		HW	-.294	.497	
		CBC	.578	5.841	
		 BW	5.841	231.688	
	Achefer	MC	.542	1.157	
		HL	.295	-8.126	
		BL	.963	15.502	
		HG	1.669	74.150	
		PW	.857	6.031	
		HW	1.371	23.804	
		CBC	1.068	6.530	
		 BW	6.530	295.460	
	Mecha	MC	1.069	18.371	
		HL	2.229	28.682	
		BL	5.602	92.232	
		HG	6.731	140.256	
		PW	1.485	25.158	
		HW	1.092	27.135	
		CBC	2.717	26.943	
		 BW	26.943	567.029	
	Banja	MC	1.216	41.491	
		HL	-.607	6.220	
		BL	4.625	145.985	
		HG	7.701	253.583	
		PW	1.807	44.748	
		HW	4.477	135.621	
		CBC	1.997	32.162	
		 BW	32.162	1050.451	
	Sinan	MC	2.564	31.617	
		HL	1.361	18.333	
		BL	3.791	78.263	

Covariance Matricesa,b	
sex	Location	MC	HL	BL	HG	PW	HW	
Femal	Sinan	HG	8.116	4.573	20.399	43.720	13.443	17.904	
		PW	5.214	5.163	10.999	13.443	11.526	9.058	
		HW	5.982	5.284	15.754	17.904	9.058	15.606	
		CBC	2.564	1.361	3.791	4.572	2.442	3.318	
		 BW	31.617	18.333	78.263	166.881	51.783	69.466	
	Total	MC	3.778	2.369	6.703	7.123	2.001	2.645	
		HL	2.369	21.163	4.530	-1.016	1.365	-1.949	
		BL	6.703	4.530	34.688	23.666	7.027	11.106	
		HG	7.123	-1.016	23.666	47.618	8.514	20.562	
		PW	2.001	1.365	7.027	8.514	4.873	4.628	
		HW	2.645	-1.949	11.106	20.562	4.628	23.372	
		CBC	1.093	.336	3.258	4.426	1.367	2.039	
		 BW	29.033	-3.718	96.696	193.040	34.187	83.518	

Covariance Matricesa,b	
sex	Location	CBC	 BW	
Femal	Sinan	HG	4.572	166.881	
		PW	2.442	51.783	
		HW	3.318	69.466	
		CBC	2.667	17.622	
		 BW	17.622	638.792	
	Total	MC	1.093	29.033	
		HL	.336	-3.718	
		BL	3.258	96.696	
		HG	4.426	193.040	
		PW	1.367	34.187	
		HW	2.039	83.518	
		CBC	1.814	17.985	
		 BW	17.985	786.465	

a. For split file sex=Male, the total covariance matrix has 399 degrees of freedom.	
b. For split file sex=Femal, the total covariance matrix has 799 degrees of freedom.	


Analysis 1


Summary of Canonical Discriminant Functions


Eigenvalues	
sex	Function	Eigenvalue	% of Variance	Cumulative %	Canonical Correlation	
Male	1	.869a	46.6	46.6	.682	
	2	.642a	34.4	81.0	.625	
	3	.258a	13.8	94.8	.453	
	4	.088a	4.7	99.5	.284	
	5	.010a	.5	100.0	.099	
Femal	1	.490b	40.0	40.0	.573	
	2	.461b	37.7	77.7	.562	
	3	.193b	15.7	93.4	.402	
	4	.063b	5.2	98.6	.244	
	5	.017b	1.4	100.0	.129	

a. For split file sex=Male, first 5 canonical discriminant functions were used in the analysis.	
b. For split file sex=Femal, first 5 canonical discriminant functions were used in the analysis.	


Wilks' Lambda	
sex	Test of Function(s)	Wilks' Lambda	Chi-square	df	Sig.	
Male	1 through 5	.236	566.243	40	.000	
	2 through 5	.441	321.008	28	.000	
	3 through 5	.724	126.645	18	.000	
	4 through 5	.910	36.777	10	.000	
	5	.990	3.881	4	.422	
Femal	1 through 5	.356	817.043	40	.000	
	2 through 5	.531	501.426	28	.000	
	3 through 5	.776	201.337	18	.000	
	4 through 5	.925	61.835	10	.000	
	5	.983	13.245	4	.010	


Standardized Canonical Discriminant Function Coefficients	
sex	Function	
	1	2	3	4	5	
Male	MC	-.002	.756	.953	-1.167	-.051	
	HL	.758	-.094	.029	.206	-.340	
	BL	-.248	-.338	-1.481	-.674	-.233	
	HG	-1.533	1.180	1.649	-1.210	-3.539	
	PW	.669	.400	.072	-.023	1.237	
	HW	-.105	-.794	.909	.040	.431	
	CBC	-.002	.466	-.079	.782	-.414	
	 BW	.486	-1.126	-1.976	2.292	2.910	
Femal	MC	.032	.867	.386	.077	.334	
	HL	-.361	.627	-.090	.153	-.036	
	BL	-.197	-.211	-.875	.179	.098	
	HG	2.843	1.698	-9.103	-5.812	6.271	
	PW	-.063	-.453	-.186	.325	-.627	
	HW	.439	.666	.183	-.725	-.456	
	CBC	-.452	-.408	.547	-.464	.619	
	 BW	-1.989	-1.983	9.233	6.547	-5.882	


Structure Matrix


For sex=Male	
sex	Function	
	1	2	3	4	5	
Male	HG	-.611*	.380	-.025	.172	-.048	
	 BW	-.611*	.365	-.026	.211	-.013	
	HW	-.559*	-.124	.249	.077	.129	
	BL	-.468*	.237	-.395	-.205	-.007	
	CBC	-.277	.600*	-.032	.383	-.054	
	MC	-.422	.590*	.198	-.228	-.102	
	PW	-.201	.569*	-.170	.074	.492	
	HL	.420	.096	.021	.091	-.509*	

Pooled within-groups correlations between discriminating variables and standardized canonical discriminant functions 
 Variables ordered by absolute size of correlation within function.	
*. Largest absolute correlation between each variable and any discriminant function	


For sex=Femal	
sex	Function	
	1	2	3	4	5	
Femal	HG	.696*	.027	.078	.414	.378	
	 BW	.683*	.024	.124	.448	.346	
	HW	.659*	.257	.136	-.254	-.184	
	HL	-.406	.582*	-.119	.326	.031	
	BL	.186	.085	-.312	.406*	.260	
	PW	.234	-.058	-.129	.311*	-.061	
	CBC	.021	-.146	.333	-.069	.580*	
	MC	.165	.458	.126	.402	.474*	

Pooled within-groups correlations between discriminating variables and standardized canonical discriminant functions 
 Variables ordered by absolute size of correlation within function.	
*. Largest absolute correlation between each variable and any discriminant function	


Functions at Group Centroids	
sex	Location	Function	
		1	2	3	4	5	
Male	Jawi	-1.484	-.249	-.171	.192	-.105	
	Enebsie	1.050	-.283	-.818	.149	.016	
	Achefer	-.167	-.567	-.078	-.744	-.009	
	Mecha	-.833	-.039	.182	.108	.241	
	Banja	.847	-.667	.781	.142	-.039	
	Sinan	.254	1.603	.138	-.059	-.028	
Femal	Jawi	.986	-.829	.352	.096	-.024	
	Enebsie	-.047	-.218	-.854	.135	-.045	
	Achefer	.081	.104	-.055	-.645	-.074	
	Mecha	.005	.225	-.025	-.043	.339	
	Banja	.248	1.226	.206	.154	-.073	
	Sinan	-1.245	-.399	.349	.073	-.034	

Unstandardized canonical discriminant functions evaluated at group means	


Classification Statistics


Classification Processing Summary	
Male	Processed	400	
	Excluded	Missing or out-of-range group codes	0	
		At least one missing discriminating variable	0	
	Used in Output	400	
Femal	Processed	800	
	Excluded	Missing or out-of-range group codes	0	
		At least one missing discriminating variable	0	
	Used in Output	800	


Prior Probabilities for Groups	
sex	Location	Prior	Cases Used in Analysis	
			Unweighted	Weighted	
Male	Jawi	.167	75	75.000	
	Enebsie	.167	75	75.000	
	Achefer	.167	50	50.000	
	Mecha	.167	50	50.000	
	Banja	.167	75	75.000	
	Sinan	.167	75	75.000	
	Total	1.000	400	400.000	
Femal	Jawi	.167	150	150.000	
	Enebsie	.167	150	150.000	
	Achefer	.167	100	100.000	
	Mecha	.167	100	100.000	
	Banja	.167	150	150.000	
	Sinan	.167	150	150.000	
	Total	1.000	800	800.000	


Territorial Maps


                             Territorial Map for sex:             1   Male
                          (Assuming all functions but the first two are zero)
Canonical Discriminant
Function 2
       -8.0      -6.0      -4.0      -2.0        .0       2.0       4.0       6.0       8.0
          +---------+---------+---------+---------+---------+---------+---------+---------+
     8.0 +                                                                                 +
         I6                                                                                I
         I166                                                                              I
         I 1166                                                                            I
         I   1166                                                                          I
         I     1166                                                                        I
     6.0 +       116+         +         +         +         +         +         +          +
         I         166                                                                     I
         I          1166                                                                   I
         I            1166                                                                 I
         I              116                                                                I
         I                166                                                              I
     4.0 +          +      1166         +         +         +         +         +          +
         I                   1166                                                         6I
         I                     1166                                                   66662I
         I                       116                                              66662222 I
         I                         166                                        66662222     I
         I                          1166                                  66662222         I
     2.0 +          +         +       1166        +         +         66662222  +          +
         I                              1166       *              66662222                 I
         I                                1466                66662222                     I
         I                                1444666         66662222                         I
         I                                 14 44466   66662222                             I
         I                                 14    446662222                                 I
      .0 +          +         +         +   14*   +432      +         +         +          +
         I                                 *14   443352*2                                  I
         I                                   14 4*3 35*55222                               I
         I                                   1443   35   555222                            I
         I                                    13    35      555222                         I
         I                                   13    35          555222                      I
    -2.0 +          +         +         +    13   +35       +     555222        +          +
         I                                   13    35                5552222               I
         I                                  13     35                   5555222            I
         I                                  13     35                       555222         I
         I                                 13      35                          555222      I
         I                                 13     35                              555222   I
    -4.0 +          +         +         +  13     35        +         +         +    555222+
         I                                13      35                                    555I
         I                                13      35                                       I
         I                               13       35                                       I
         I                               13       35                                       I
         I                               13      35                                        I
    -6.0 +          +         +         13       35         +         +         +          +
         I                              13       35                                        I
         I                             13        35                                        I
         I                             13        35                                        I
         I                             13        35                                        I
         I                            13        35                                         I
    -8.0 +                            13        35                                         +
          +---------+---------+---------+---------+---------+---------+---------+---------+
       -8.0      -6.0      -4.0      -2.0        .0       2.0       4.0       6.0       8.0
                                   Canonical Discriminant Function 1


Symbols used in territorial map

Symbol  Group  Label
------  -----  --------------------

   1        1  Jawi
   2        2  Enebsie
   3        3  Achefer
   4        4  Mecha
   5        5  Banja
   6        6  Sinan
   *           Indicates a group centroid


                            Territorial Map for sex:             2   Femal
                          (Assuming all functions but the first two are zero)
Canonical Discriminant
Function 2
       -8.0      -6.0      -4.0      -2.0        .0       2.0       4.0       6.0       8.0
          +---------+---------+---------+---------+---------+---------+---------+---------+
     8.0 +                                                                                 +
         I                                                                                 I
         I55                                                                               I
         I665                                                                              I
         I  655                                                                            I
         I   6655                                                                          I
     6.0 +     6655 +         +         +         +         +         +         +          +
         I       6655                                                                      I
         I         6655                                                                    I
         I           665                                                                   I
         I             655                                                                 I
         I              6655                                                               I
     4.0 +          +     6655+         +         +         +         +         +          +
         I                  6655                                                           I
         I                    665                                                          I
         I                      655                                                        I
         I                       6655                                                 55555I
         I                         6655                                           555511111I
     2.0 +          +         +      6655         +         +         +      555551111     +
         I                             6655                             5555511111         I
         I                               665       *                555511111              I
         I                                 655555              555551111                   I
         I                                  664445555555  5555511111                       I
         I                                   64  4*444445511111                            I
      .0 +          +         +         +     64  *   4411  +         +         +          +
         I                                  *  64 * 4411                                   I
         I                                      644411 *                                   I
         I                                      6221                                       I
         I                                       61                                        I
         I                                       61                                        I
    -2.0 +          +         +         +       61+         +         +         +          +
         I                                      61                                         I
         I                                      61                                         I
         I                                     61                                          I
         I                                     61                                          I
         I                                     61                                          I
    -4.0 +          +         +         +     61  +         +         +         +          +
         I                                    61                                           I
         I                                    61                                           I
         I                                   61                                            I
         I                                   61                                            I
         I                                   61                                            I
    -6.0 +          +         +         +   61    +         +         +         +          +
         I                                  61                                             I
         I                                  61                                             I
         I                                 61                                              I
         I                                 61                                              I
         I                                 61                                              I
    -8.0 +                                61                                               +
          +---------+---------+---------+---------+---------+---------+---------+---------+
       -8.0      -6.0      -4.0      -2.0        .0       2.0       4.0       6.0       8.0
                                   Canonical Discriminant Function 1


Symbols used in territorial map

Symbol  Group  Label
------  -----  --------------------

   1        1  Jawi
   2        2  Enebsie
   3        3  Achefer
   4        4  Mecha
   5        5  Banja
   6        6  Sinan
   *           Indicates a group centroid


Separate-Groups Graphs


sex=Male


sex=Femal


All-Groups Scatter Plots
