# Supplementary material for: Quantifying morphometric and adaptive characteristics of indigenous cattle genetic resources in northwest Ethiopia
Source: PLoS One. 2023 Mar 20;18(3):e0280640. doi: 10.1371/journal.pone.0280640 (PMC10027228; doi:10.1371/journal.pone.0280640)
Supplement: S5 File — (DOC) [file pone.0280640.s005.doc]

SPLIT FILE OFF.
DISCRIMINANT
  /GROUPS=Location(1 6)
  /VARIABLES=MC HL BL HG PW HW CBC BW
  /ANALYSIS ALL
  /PRIORS EQUAL
  /STATISTICS=UNIVF GCOV TCOV
  /PLOT=COMBINED SEPARATE  MAP
  /CLASSIFY=NONMISSING POOLED.


Discriminant


Notes	
Output Created	09-DEC-2022 17:30:25	
Comments		
Input	Data	C:\Users\DEMSSIE\Desktop\data final\Untitled3.sav	
	Active Dataset	DataSet1	
	Filter	<none>	
	Weight	<none>	
	Split File	<none>	
	N of Rows in Working Data File	1200	
Missing Value Handling	Definition of Missing	User-defined missing values are treated as missing in the analysis phase.	
	Cases Used	In the analysis phase, cases with no user- or system-missing values for any predictor variable are used. Cases with user-, system-missing, or out-of-range values for the grouping variable are always excluded.	
Syntax	DISCRIMINANT
  /GROUPS=Location(1 6)
  /VARIABLES=MC HL BL HG PW HW CBC BW
  /ANALYSIS ALL
  /PRIORS EQUAL
  /STATISTICS=UNIVF GCOV TCOV
  /PLOT=COMBINED SEPARATE  MAP
  /CLASSIFY=NONMISSING POOLED.	
Resources	Processor Time	00:00:01.20	
	Elapsed Time	00:00:01.18	


[DataSet1] C:\Users\DEMSSIE\Desktop\data final\Untitled3.sav


Analysis Case Processing Summary	
Unweighted Cases	N	Percent	
Valid	1200	100.0	
Excluded	Missing or out-of-range group codes	0	.0	
	At least one missing discriminating variable	0	.0	
	Both missing or out-of-range group codes and at least one missing discriminating variable	0	.0	
	Total	0	.0	
Total	1200	100.0	


Group Statistics	
Location	Valid N (listwise)	
	Unweighted	Weighted	
Jawi	MC	225	225.000	
	HL	225	225.000	
	BL	225	225.000	
	HG	225	225.000	
	PW	225	225.000	
	HW	225	225.000	
	CBC	225	225.000	
	 BW	225	225.000	
Enebsie	MC	225	225.000	
	HL	225	225.000	
	BL	225	225.000	
	HG	225	225.000	
	PW	225	225.000	
	HW	225	225.000	
	CBC	225	225.000	
	 BW	225	225.000	
Achefer	MC	150	150.000	
	HL	150	150.000	
	BL	150	150.000	
	HG	150	150.000	
	PW	150	150.000	
	HW	150	150.000	
	CBC	150	150.000	
	 BW	150	150.000	
Mecha	MC	150	150.000	
	HL	150	150.000	
	BL	150	150.000	
	HG	150	150.000	
	PW	150	150.000	
	HW	150	150.000	
	CBC	150	150.000	
	 BW	150	150.000	

Group Statistics	
Location	Valid N (listwise)	
	Unweighted	Weighted	
Banja	MC	225	225.000	
	HL	225	225.000	
	BL	225	225.000	
	HG	225	225.000	
	PW	225	225.000	
	HW	225	225.000	
	CBC	225	225.000	
	 BW	225	225.000	
Sinan	MC	225	225.000	
	HL	225	225.000	
	BL	225	225.000	
	HG	225	225.000	
	PW	225	225.000	
	HW	225	225.000	
	CBC	225	225.000	
	 BW	225	225.000	
Total	MC	1200	1200.000	
	HL	1200	1200.000	
	BL	1200	1200.000	
	HG	1200	1200.000	
	PW	1200	1200.000	
	HW	1200	1200.000	
	CBC	1200	1200.000	
	 BW	1200	1200.000	


Tests of Equality of Group Means	
	Wilks' Lambda	F	df1	df2	Sig.	
MC	.963	9.149	5	1194	.000	
HL	.843	44.447	5	1194	.000	
BL	.972	6.923	5	1194	.000	
HG	.892	29.041	5	1194	.000	
PW	.972	6.854	5	1194	.000	
HW	.850	42.044	5	1194	.000	
CBC	.942	14.617	5	1194	.000	
 BW	.892	29.040	5	1194	.000	


Covariance Matricesa	
Location	MC	HL	BL	HG	PW	HW	CBC	
Jawi	MC	10.498	6.407	19.994	24.867	3.185	10.423	3.639	
	HL	6.407	24.775	12.934	19.033	2.567	5.848	2.784	
	BL	19.994	12.934	74.899	64.444	10.757	27.523	9.041	
	HG	24.867	19.033	64.444	98.631	13.516	41.166	11.559	
	PW	3.185	2.567	10.757	13.516	4.858	5.196	1.679	
	HW	10.423	5.848	27.523	41.166	5.196	25.550	5.219	
	CBC	3.639	2.784	9.041	11.559	1.679	5.219	3.096	
	 BW	117.351	86.891	295.648	462.675	61.334	193.631	53.234	
Enebsie	MC	3.384	1.748	3.877	7.442	1.019	-.296	.829	
	HL	1.748	15.341	3.107	4.656	.964	-1.679	.988	
	BL	3.877	3.107	10.825	11.104	3.041	2.705	1.567	
	HG	7.442	4.656	11.104	29.131	1.123	7.053	1.564	
	PW	1.019	.964	3.041	1.123	2.005	-.306	.221	
	HW	-.296	-1.679	2.705	7.053	-.306	21.362	-.167	
	CBC	.829	.988	1.567	1.564	.221	-.167	1.223	
	 BW	29.577	18.570	43.971	115.457	4.175	28.045	6.571	
Achefer	MC	5.454	2.070	9.745	10.897	1.735	3.878	2.038	
	HL	2.070	28.826	4.963	.948	.120	-.755	.442	
	BL	9.745	4.963	34.343	26.484	4.381	9.203	4.457	
	HG	10.897	.948	26.484	47.486	7.023	15.789	6.234	
	PW	1.735	.120	4.381	7.023	3.892	3.948	1.552	
	HW	3.878	-.755	9.203	15.789	3.948	15.073	2.571	
	CBC	2.038	.442	4.457	6.234	1.552	2.571	1.729	
	 BW	46.532	3.330	112.136	198.890	29.475	65.368	26.473	
Mecha	MC	6.440	.475	11.636	21.927	3.911	8.828	3.616	
	HL	.475	20.928	10.391	4.654	2.595	-1.473	1.228	
	BL	11.636	10.391	48.190	52.440	11.454	21.506	9.033	
	HG	21.927	4.654	52.440	98.804	17.596	39.964	15.146	
	PW	3.911	2.595	11.454	17.596	5.543	6.783	2.746	
	HW	8.828	-1.473	21.506	39.964	6.783	33.290	5.932	
	CBC	3.616	1.228	9.033	15.146	2.746	5.932	3.546	
	 BW	100.445	17.280	234.274	445.132	79.420	183.133	67.540	
Banja	MC	3.354	1.196	9.009	9.391	2.288	4.588	1.595	

Covariance Matricesa	
Location	 BW	
Jawi	MC	117.351	
	HL	86.891	
	BL	295.648	
	HG	462.675	
	PW	61.334	
	HW	193.631	
	CBC	53.234	
	 BW	2192.311	
Enebsie	MC	29.577	
	HL	18.570	
	BL	43.971	
	HG	115.457	
	PW	4.175	
	HW	28.045	
	CBC	6.571	
	 BW	458.233	
Achefer	MC	46.532	
	HL	3.330	
	BL	112.136	
	HG	198.890	
	PW	29.475	
	HW	65.368	
	CBC	26.473	
	 BW	835.626	
Mecha	MC	100.445	
	HL	17.280	
	BL	234.274	
	HG	445.132	
	PW	79.420	
	HW	183.133	
	CBC	67.540	
	 BW	2019.160	
Banja	MC	39.145	

Covariance Matricesa	
Location	MC	HL	BL	HG	PW	HW	CBC	
Banja	HL	1.196	17.307	.787	-1.595	-.641	-2.414	-.336	
	BL	9.009	.787	49.921	37.049	11.234	22.091	3.899	
	HG	9.391	-1.595	37.049	55.186	12.032	27.680	6.283	
	PW	2.288	-.641	11.234	12.032	5.193	6.900	1.330	
	HW	4.588	-2.414	22.091	27.680	6.900	24.464	4.259	
	CBC	1.595	-.336	3.899	6.283	1.330	4.259	2.384	
	 BW	39.145	-5.599	152.460	227.460	49.389	113.949	26.614	
Sinan	MC	16.450	5.915	21.293	36.938	9.182	14.610	7.069	
	HL	5.915	9.703	8.448	12.140	4.581	6.965	2.294	
	BL	21.293	8.448	39.956	52.534	15.110	24.815	9.356	
	HG	36.938	12.140	52.534	118.168	26.669	40.973	17.902	
	PW	9.182	4.581	15.110	26.669	13.215	11.012	4.814	
	HW	14.610	6.965	24.815	40.973	11.012	24.439	7.746	
	CBC	7.069	2.294	9.356	17.902	4.814	7.746	5.126	
	 BW	156.895	52.174	220.779	496.009	110.970	172.605	75.740	
Total	MC	8.069	3.099	12.726	19.463	3.681	7.332	3.313	
	HL	3.099	22.209	4.907	2.334	1.278	-.893	.924	
	BL	12.726	4.907	44.311	43.202	9.798	19.114	6.297	
	HG	19.463	2.334	43.202	83.487	13.728	33.433	10.264	
	PW	3.681	1.278	9.798	13.728	6.063	5.507	2.174	
	HW	7.332	-.893	19.114	33.433	5.507	28.120	4.187	
	CBC	3.313	.924	6.297	10.264	2.174	4.187	3.041	
	 BW	85.632	10.338	187.162	362.628	58.956	145.430	45.025	

Covariance Matricesa	
Location	 BW	
Banja	HL	-5.599	
	BL	152.460	
	HG	227.460	
	PW	49.389	
	HW	113.949	
	CBC	26.614	
	 BW	943.847	
Sinan	MC	156.895	
	HL	52.174	
	BL	220.779	
	HG	496.009	
	PW	110.970	
	HW	172.605	
	CBC	75.740	
	 BW	2091.720	
Total	MC	85.632	
	HL	10.338	
	BL	187.162	
	HG	362.628	
	PW	58.956	
	HW	145.430	
	CBC	45.025	
	 BW	1589.381	

a. The total covariance matrix has 1199 degrees of freedom.	


Analysis 1


Summary of Canonical Discriminant Functions


Eigenvalues	
Function	Eigenvalue	% of Variance	Cumulative %	Canonical Correlation	
1	.449a	49.5	49.5	.557	
2	.314a	34.7	84.2	.489	
3	.120a	13.2	97.4	.327	
4	.020a	2.2	99.6	.139	
5	.004a	.4	100.0	.063	

a. First 5 canonical discriminant functions were used in the analysis.	


Wilks' Lambda	
Test of Function(s)	Wilks' Lambda	Chi-square	df	Sig.	
1 through 5	.458	930.586	40	.000	
2 through 5	.664	488.341	28	.000	
3 through 5	.872	162.648	18	.000	
4 through 5	.977	28.036	10	.002	
5	.996	4.681	4	.322	


Standardized Canonical Discriminant Function Coefficients	
	Function	
	1	2	3	4	5	
MC	.580	.615	.968	-.401	.707	
HL	.572	.431	-.139	.615	-.062	
BL	-.047	-.356	-1.041	-.340	.953	
HG	-1.962	3.193	-3.046	.354	1.219	
PW	.267	-.363	-.074	.180	-.494	
HW	-.377	1.068	.185	-.401	-.183	
CBC	.240	-.636	.307	-.433	-.132	
 BW	.906	-3.724	3.125	1.070	-1.439	


Structure Matrix	
	Function	
	1	2	3	4	5	
HW	-.537*	.347	.268	.073	.267	
CBC	-.056	-.326	.464*	.040	.373	
HL	.575	.305	-.060	.629*	.188	
 BW	-.453	-.145	.368	.532*	.484	
HG	-.466	-.123	.328	.531*	.492	
PW	-.082	-.271	.054	.333*	.170	
BL	-.194	-.149	-.125	.180	.811*	
MC	-.015	-.017	.540	.238	.731*	

Pooled within-groups correlations between discriminating variables and standardized canonical discriminant functions 
 Variables ordered by absolute size of correlation within function.	
*. Largest absolute correlation between each variable and any discriminant function	


Functions at Group Centroids	
Location	Function	
	1	2	3	4	5	
Jawi	-1.193	-.346	.129	.057	-.042	
Enebsie	.253	-.200	-.678	.062	-.001	
Achefer	-.154	.418	-.080	-.352	-.015	
Mecha	-.192	-.008	.163	.019	.162	
Banja	.294	.959	.165	.122	-.029	
Sinan	.877	-.687	.329	-.019	-.026	

Unstandardized canonical discriminant functions evaluated at group means	


Classification Statistics


Classification Processing Summary	
Processed	1200	
Excluded	Missing or out-of-range group codes	0	
	At least one missing discriminating variable	0	
Used in Output	1200	


Prior Probabilities for Groups	
Location	Prior	Cases Used in Analysis	
		Unweighted	Weighted	
Jawi	.167	225	225.000	
Enebsie	.167	225	225.000	
Achefer	.167	150	150.000	
Mecha	.167	150	150.000	
Banja	.167	225	225.000	
Sinan	.167	225	225.000	
Total	1.000	1200	1200.000	


                                            Territorial Map
                          (Assuming all functions but the first two are zero)
Canonical Discriminant
Function 2
       -8.0      -6.0      -4.0      -2.0        .0       2.0       4.0       6.0       8.0
          +---------+---------+---------+---------+---------+---------+---------+---------+
     8.0 +   155                                                                           +
         I    115                                                                          I
         I      155                                                                        I
         I       115                                                                       I
         I         155                                                                     I
         I          115                                                                    I
     6.0 +          + 155     +         +         +         +         +         +          +
         I             115                                                                 I
         I               15                                                                I
         I                155                                                              I
         I                 115                                                             I
         I                   155                                                           I
     4.0 +          +         115       +         +         +         +         +          +
         I                      155                                                        I
         I                       115                                                       I
         I                         155                                                     I
         I                          1155                                                555I
         I                            1355                                         55555666I
     2.0 +          +         +        13355      +         +         +       5555566666   +
         I                              133355                           5555566666        I
         I                               1133355                     555566666             I
         I                                 13 3355 *            555556666                  I
         I                                  13  3355       5555566666                      I
         I                                   1333*33555555566666                           I
      .0 +          +         +         +     144*444426666 +         +         +          +
         I                                  *  14  *466                                    I
         I                                     14  46 *                                    I
         I                                      1446                                       I
         I                                      146                                        I
         I                                       16                                        I
    -2.0 +          +         +         +       16+         +         +         +          +
         I                                      16                                         I
         I                                      16                                         I
         I                                     16                                          I
         I                                     16                                          I
         I                                     16                                          I
    -4.0 +          +         +         +      16 +         +         +         +          +
         I                                    16                                           I
         I                                    16                                           I
         I                                    16                                           I
         I                                   16                                            I
         I                                   16                                            I
    -6.0 +          +         +         +    16   +         +         +         +          +
         I                                   16                                            I
         I                                  16                                             I
         I                                  16                                             I
         I                                  16                                             I
         I                                  16                                             I
    -8.0 +                                 16                                              +
          +---------+---------+---------+---------+---------+---------+---------+---------+
       -8.0      -6.0      -4.0      -2.0        .0       2.0       4.0       6.0       8.0
                                   Canonical Discriminant Function 1


Symbols used in territorial map

Symbol  Group  Label
------  -----  --------------------

   1        1  Jawi
   2        2  Enebsie
   3        3  Achefer
   4        4  Mecha
   5        5  Banja
   6        6  Sinan
   *           Indicates a group centroid


Separate-Groups Graphs
